# Congruence Gaps Between Adolescents With Cancer and Their Families Regarding Values, Goals, and Beliefs About End-of-Life Care

**DOI:** 10.1001/jamanetworkopen.2020.5424

**Published:** 2020-05-19

**Authors:** Sarah Friebert, Daniel H. Grossoehme, Justin N. Baker, Jennifer Needle, Jessica D. Thompkins, Yao I. Cheng, Jichuan Wang, Maureen E. Lyon

**Affiliations:** 1 Haslinger Family Pediatric Palliative Care Center, Akron Children’s Hospital, Akron, Ohio; 2 Rebecca D. Considine Research Institute, Akron Children’s Hospital, Akron, Ohio; 3 Division of Quality of Life and Palliative Care, St Jude Research Hospital, Memphis, Tennessee; 4 Center for Bioethics, Department of Pediatrics, University of Minnesota, Minneapolis; 5 Center for Translational Research, Children’s Research Institute, Children’s National Hospital, Washington, DC; 6 Division of Biostatistics and Study Methodology, Center for Translational Research, Children’s Research Institute, Children’s National Hospital, Washington, DC; 7 George Washington University School of Medicine and Health Sciences, Washington, DC; 8 Division of Adolescent and Young Adult Medicine, Center for Translational Research, Children’s Research Institute, Children’s National Hospital, Washington, DC

## Abstract

**Importance:**

Lack of pediatric advance care planning has been associated with poor communication, increased hospitalization, poor quality of life, and legal actions. Clinicians presume that families understand adolescents’ treatment preferences for end-of-life care.

**Objective:**

To examine patient-reported end-of-life values and needs of adolescents with cancer and congruence with their families’ understanding of these needs.

**Design, Setting, and Participants:**

This cross-sectional survey was conducted among adolescent-family dyads from July 16, 2016, to April 30, 2019, at 4 tertiary care pediatric US hospitals. Participants included 80 adolescent-family dyads (160 participants) within a larger study facilitating pediatric advance care planning. Adolescent eligibility criteria included being aged 14 to 21 years, English speaking, being diagnosed with cancer at any stage, and knowing their diagnosis. Family included legal guardians for minors or chosen surrogate decision-makers for those aged 18 years or older. Data analysis was performed from April 2019 to November 2019.

**Exposure:**

Session 1 of the 3-session Family Centered Pediatric Advance Care Planning for Teens With Cancer intervention.

**Main Outcomes and Measures:**

The main outcome was congruence between adolescents with cancer and their families regarding adolescents’ values, goals, and beliefs about end-of-life care. Prevalence-adjusted and bias-adjusted κ (PABAK) values were used to measure congruence on the Lyon Advance Care Planning Survey-Revised (Patient and Surrogate versions).

**Results:**

A total of 80 adolescent-family dyads (160 participants) were randomized to the intervention group in the original trial. Among the adolescents, 44 (55.0%) were female and 60 (75.0%) were white, with a mean (SD) age of 16.9 (1.8) years. Among family members, 66 (82.5%) were female and 65 (81.3%) were white, with a mean (SD) age of 45.3 (8.3) years. Family members’ understanding of their adolescent’s beliefs about the best time bring up end-of-life decisions was poor: 86% of adolescents wanted early timing (before getting sick, while healthy, when first diagnosed, when first sick from a life-threatening illness, or all of the above), but only 39% of families knew this (PABAK, 0.18). Families’ understanding of what was important to their adolescents when dealing with their own dying was excellent for wanting honest answers from their physician (PABAK, 0.95) and understanding treatment choices (PABAK, 0.95) but poor for dying a natural death (PABAK, 0.18) and being off machines that extend life, if dying (PABAK, 0).

**Conclusions and Relevance:**

Many families had a poor understanding of their adolescent’s values regarding their own end-of-life care, such as when to initiate end-of-life conversations and preference for being off machines that extend life. Pediatric advance care planning could minimize these misunderstandings with the potential for a substantial impact on quality of care.

## Key Points

**Question:**

Do families of adolescents with cancer know what the adolescent would want if they were dying?

**Findings:**

In this cross-sectional study of 80 adolescent-family dyads (160 participants), critical congruence gaps were found between adolescents with cancer and their families. Families had a poor understanding of their adolescents’ preferences for the best time to bring up end-of-life decisions, dying a natural death, and being off life support if they were dying, but families’ understanding of adolescents’ relational needs was excellent.

**Meaning:**

These findings suggest that family-centered pediatric advance care planning interventions are needed to close the gaps in families’ knowledge of adolescents’ end-of-life treatment preferences.

## Teaser

This cross-sectional study examines patient-reported end-of-life values and needs of adolescents with cancer and congruence with their families’ understanding of these needs.

## Introduction

Cancer remains the leading cause of disease-related death for adolescents.^1,2^ For adolescent patients with cancer, death frequently occurs in the context of withholding and withdrawing life-sustaining treatment.^3,4^ If parents are being asked to make these difficult decisions, a prior understanding of their child’s preferences may ease the burden of decision-making. Unfortunately, the timing of these discussions is often very close to death, preventing thoughtful reflection about how these decisions affect the patient and their family.^3,4^

Preparation for the possibility of death includes open and honest communication between adolescents with cancer and their families. Pediatric advance care planning (pACP) is the reference standard in the care of patients with life-limiting illnesses^5^ and is internationally recognized as a need by clinicians.^6,7,8^ Small pilot and qualitative studies^9,10,11^ show that parents desire to have pACP conversations and to keep all options open. Nevertheless, policy recommendations^12,13^ to include adolescents in shared decision-making remain aspirational, despite findings from qualitative and pilot studies^14,15,16,17,18^ showing that adolescents with cancer want to engage in pACP. Most adolescents aged 14 years and older do not differ from adults in their capacity to make informed treatment decisions, and their understanding of death is no less mature than that of adults.^15,19^

Benefits of pACP, as identified in the pilot trial^20^ (30 dyads) of the current larger scale study, include families’ improved understanding of their adolescent’s treatment preferences and adolescents receiving earlier palliative care. Trials of the same pACP intervention with adolescents with HIV (105 dyads) also demonstrated improved congruence on end-of-life (EOL) preferences^21,22^ and decreased HIV-specific symptoms among adolescents longitudinally.^22^ Among children with complex chronic conditions, pACP decreased suffering at the EOL and improved families’ quality of life.^23^ Lack of ACP has been associated with poor communication, increased hospitalization, poor EOL quality of life, poorer adherence to patient’s EOL preferences, and legal actions.^24,25,26^

Conversations about hoping for the best while planning for the worst are emotional and often are avoided or considered taboo.^17^ Structured patient-centered and family-supported pACP conducted by trained or certified facilitators safely elicited strong emotions among HIV-positive adolescents and their families.^27^ It remains unknown whether early pACP builds families’ capacity to make EOL decisions for their child, which could allay clinicians’ concerns about burdening parents^7,28,29^ or their lack of readiness.^30^ The discomfort reported by clinicians about knowing what to say, especially when it comes to discussing resuscitation status,^6,30,31^ may be minimized, if this is not the first time the adolescent and/or family has had this conversation. Parents of children with cancer define being a good parent to include making informed, unselfish decisions in the child’s best interest and teaching their child to make good decisions.^16^ Parental perspectives on pACP focus on what a loving parent would do.^31^ Yet, few systematic pACP programs exist,^32,33^ with only 1 model tested in a fully powered randomized clinical trial.^21,22^ Most studies on pACP presume that families accurately represent the adolescent patient’s goals, values, and EOL treatment preferences.^5,7,31^ Only our small pilot study^8^ from a single site has empirically tested this presumption for adolescents with cancer (17 adolescent-family dyads) and found it to be false. The current multisite cross-sectional study of pACP enrolled the largest sample of adolescents with cancer and their families to date, adding geographical and economic diversity to the pilot. We identified key areas of misunderstanding during session 1 of a 3-session pACP intervention, so as to close gaps in understanding during pACP conversations during sessions 2 and 3.

## Methods

### Study Design and Participants

**Figure 1.**
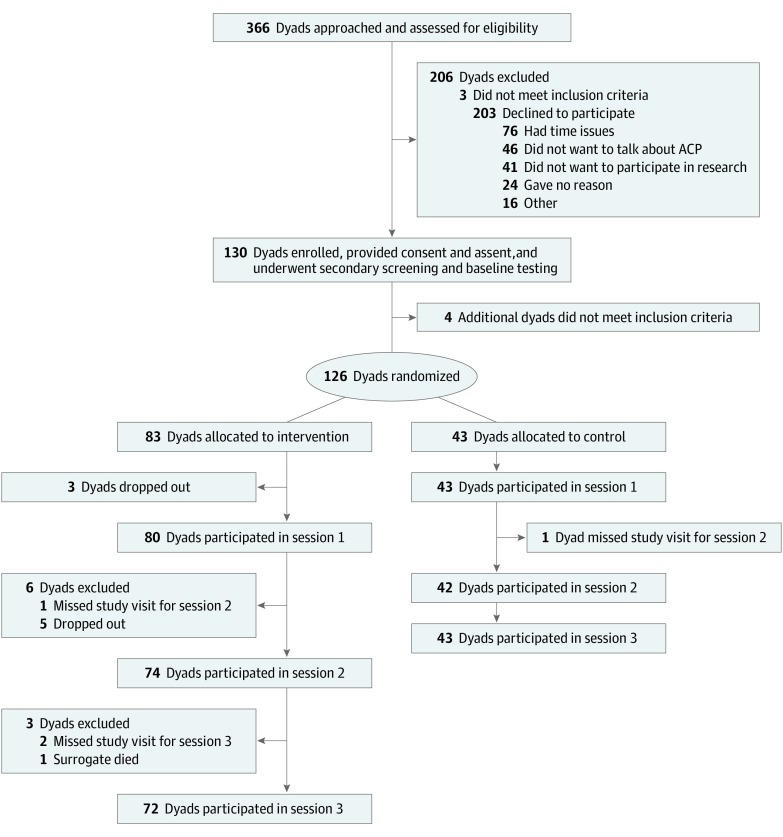
CONSORT Diagram for the Original Family Centered Pediatric Advance Care Planning for Teens With Cancer Trial ACP indicates advance care planning.

The Family Centered Pediatric Advance Care Planning for Teens With Cancer (FACE-TC) intervention is a 2-group randomized clinical trial designed to evaluate the efficacy of an adequately powered pACP intervention (Figure 1). For this study, the term *family* refers to the legal guardian for adolescents aged 14 to 17 years or surrogate decision-makers for adolescents aged 18 to 21 years chosen by the adolescents themselves. The design, methods, and power analysis for determining a sample size of 130 enrolled dyads are published elsewhere.^34^

Between July 16, 2016, and April 30, 2019, adolescents with cancer and their families were recruited from 4 tertiary care pediatric hospitals: Akron Children’s Hospital (Akron, Ohio), St Jude Children’s Research Hospital (Memphis, Tennessee), University of Minnesota Masonic Children’s Hospital (Minneapolis, Minnesota), and Children’s National Hospital (Washington, DC). Inclusion criteria for adolescents were a diagnosis of any cancer at any stage; awareness of the diagnosis; age 14 to 21 years; English speaking; not developmentally delayed, depressed, homicidal, suicidal, or psychotic; and not in foster care. Inclusion and exclusion criteria for families were similar, except the family member had to be aged 18 years or older.

The trial was reviewed and approved by institutional review boards from each site. Written informed consent and assent were obtained for all participants. This study follows the Strengthening the Reporting of Observational Studies in Epidemiology (STROBE) reporting guideline.

### Procedure

After consulting with a patient’s primary oncology physician, research assistants approached potentially eligible participants face-to-face during hospital outpatient visits and stays. The trial comprises 8 visits over 2 years. The first visit included enrollment, secondary screening, and, if eligible, enrollment and completion of baseline questionnaires. Dyads were then randomized to the FACE-TC intervention or treatment as usual control, using a computerized 2:1 ratio. In the FACE-TC group, session 1 included the Lyon FACE-TC ACP Survey, session 2 included the Next Steps: Respecting Choices Interview,^35^ and session 3 included completion of advance directives (Five Wishes survey).^36^ Ongoing visits measuring outcomes occurred at 3, 6, 12, and 18 months after the intervention. Results reported here are from the survey conducted in session 1. The primary outcome of the trial is longitudinal congruence in treatment preferences about 5 cancer-related situations, as measured by the Statement of Treatment Preferences administered to intervention and control dyads immediately after session 2 and at 3, 6, 12, and 18 months after the intervention.^34,35^

### Data Source and Measures

The Demographic Data Form was administered by a trained research assistant at baseline to obtain patient-reported sociodemographic information. Medical data were obtained by the research assistant through medical record review and abstraction.

The Lyon Advance Care Planning Survey-Revised (Patient and Surrogate versions) is a 31-item instrument developed and adapted with permission on the basis of an integration of 3 evidence-based surveys^37,38,39^ seamlessly interwoven into a highly structured questionnaire. The Flesch-Kincaid Grade Level for reading is 6.8. It has demonstrated acceptability and feasibility. The survey was administered face-to-face, individually in a private room, by facilitators trained to minimize social desirability bias in respondents during session 1 of the 3-session FACE-TC intervention group. As an example, the patient version poses the question, “How comfortable are you talking about death?” whereas the family version asks, “How comfortable do you think [patient’s name] is talking about death?” Responses are on a 5-point Likert scale.

### Statistical Analysis

Descriptive statistics were calculated for all variables. Pearson χ^2^ test or Fisher exact test (2-sided) was used to test response difference between age, sex, and racial groups. For congruence tests, all items were dichotomized because many items had very few cases in some categories, making it inappropriate to treat them as continuous variables. Coding used the same cutoffs as in prior studies^8,39^: 1 (very important or somewhat important) vs 0 (neither important nor unimportant, not very important, not at all important, or do not know); 1 (very comfortable or somewhat comfortable) vs 0 (neither comfortable nor uncomfortable, not very comfortable, not at all comfortable, or do not know); 1 (very concerned or concerned) vs 0 (neither concerned nor unconcerned, not concerned, not at all concerned, or do not know); and 1 (strongly agree or agree) vs 0 (neither agree nor disagree, disagree, strongly disagree, or do not know). For this exploratory analysis, comparing people who reported important with those who reported not important is reasonable. To adjust for bias in κ statistics caused by imbalanced responses, the prevalence-adjusted and bias-adjusted κ (PABAK) was calculated for assessment of agreement.^40,41^ The guidelines for the interpretation of PABAK coefficients are poor (<0.40), fair (0.40-0.59), good (0.60-0.74), and excellent (0.75-1.00).^40^

Data were entered into REDCap software version 8.10.18 (Vanderbilt University) and analyzed using SAS statistical software version 19.2 (SAS Institute). Statistical significance level was set to α = .05. When multiple tests were performed simultaneously, the Bonferroni-corrected *P* value was used as the new threshold for a single test. Data analysis was performed from April 2019 to November 2019.

## Results

### Participant Characteristics

Data presented here report on the 80 adolescent-family dyads randomized to the intervention group of the FACE-TC trial who attended session 1 (Figure 1). Adolescent participants had a mean (SD) age of 16.9 (1.8) years; family members’ mean (SD) age was 45.3 (8.3) years. Among adolescents, 55.0% (44 participants) were female, 45.0% (36 participants) were male, and 75.0% (60 participants) were white. Among their family members, 82.5% (66 participants) were female and 81.3% (65 participants) were white. Leukemia and solid tumors accounted for more than 60% of diagnoses (25 participants [31.3%] each), followed by brain tumors (16 participants [20.0%]) and lymphoma (9 participants [11.3%]). None of the adolescents had an advance directive in their medical record (Table 1). Three hundred sixty-six adolescent-family dyads were approached, of whom 336 dyads met initial eligibility criteria (Figure 1). Of these, 203 declined, 3 were ineligible, and 130 dyads enrolled (39% participation rate), achieving the predetermined sample size.^34^ Of those who declined, 23% (46 of 203 dyads) had at least 1 member of the dyad report who did not want to talk about pACP (Figure 1). The major reason given for declining was lack of time to commit to a 2-year study. The percentage of declining to participate was higher for male participants than for female participants (115 of 198 participants [58%] vs 57 of 130 participants [44%]; difference, 14%; 95% CI, 4%-25%; *P* = .02), as shown in eTable 1 in the Supplement. Age, race, ethnicity, diagnosis, and active treatment status were not statistically significantly different between those who enrolled and those who declined participation.

**Table 1.** Demographic and Clinical Characteristics of Participants at Session 1

Variable	Participants, No. (%) (N = 160)	
	Adolescents (n = 80)	Families (n = 80)
Age, mean (SD) [range], y	16.9 (1.8) [14-20]	45.3 (8.3) [19-67]
Sex		
Male	36 (45.0)	14 (17.5)
Female	44 (55.0)	66 (82.5)
Race		
Asian	3 (3.8)	3 (3.8)
Black or African American	12 (15.0)	10 (12.5)
White	60 (75.0)	65 (81.3)
> 1 Race	4 (5.0)	2 (2.5)
Declined	1 (1.3)	0
Ethnicity		
Hispanic or Latino	5 (6.3)	4 (5.0)
Not Hispanic or Latino	74 (92.5)	76 (95.0)
Declined	1 (1.3)	0
Diagnosis		
Leukemia	25 (31.3)	NA
Lymphoma	9 (11.3)	NA
Solid tumors	25 (31.3)	NA
Brain tumor	16 (20.0)	NA
Other	5 (6.3)	NA
No advance directive in medical record	80 (100.0)	NA
Relationship of families to adolescents		
Biological		
Mother	NA	60 (75.0)
Father	NA	13 (16.3)
Adoptive mother	NA	4 (5.0)
Aunt	NA	1 (1.3)
Sexual partner	NA	1 (0.8)
Girlfriend	NA	1 (0.8)

Abbreviation: NA, not applicable.

### Adolescent Preferences

Table 2 shows selected adolescent responses regarding their self-reported EOL needs. One item was missing from 1 adolescent. Otherwise, there were no missing data. The original adolescent responses to all the items are provided by age group in eTable 2 in the Supplement. Although 100% of adolescents wanted honest answers from their physicians, only 28% agreed or strongly agreed that “if someone could tell me when I would die, I would want to know.” More than 90% rated as very important or important, if dealing with their own dying, “family and friends visiting” (77 participants [96.2%]) and “understanding my treatment choices” (78 participants [97.5%]). Likewise, more than 90% rated when thinking about dying as very important or important “being physically comfortable” (73 participants [91.3%]), “saying everything I have to say to my family” (77 participants [97.5%]), “being at peace spiritually” (74 participants [92.5%]), and “having a sense of my own worth or value” (72 participants [90.0%]).

**Table 2.** Selected Responses to End-of-Life Needs Survey for Adolescents

Question	Participants, No. (%) (N = 80)
How important would each of the following be to you if you were dealing with your own dying?	
Family and friends visiting me	
Important	77 (96.2)
Not important	2 (2.5)
Don’t know	1 (1.3)
Being able to stay in my own home	
Important	61 (76.2)
Not important	18 (22.5)
Don’t know	1 (1.3)
Honest answers from my doctor	
Important	80 (100.0)
Not important	0
Don’t know	0
Comfort from church services or persons such as a minister, priest, imam, or rabbi	
Important	52 (65.0)
Not important	27 (33.8)
Don’t know	1 (1.3)
Planning my own funeral	
Important	43 (53.7)
Not important	34 (42.5)
Don’t know	3 (3.8)
Being able to complete an advance directive that would let loved ones know my wishes	
Important	71 (88.7)
Not important	4 (5.0)
Don’t know	5 (6.3)
Fulfilling personal goals or pleasure	
Important	77 (96.3)
Not important	3 (3.8)
Don’t know	0
Reviewing my life history with my family	
Important	57 (71.2)
Not important	22 (27.5)
Don’t know	1 (1.3)
Having health care professionals visit me at my home	
Important	58 (72.5)
Not important	21 (26.3)
Don’t know	1 (1.3)
Understanding my treatment choices	
Important	78 (97.5)
Not important	0
Don’t know	2 (2.5)
How important are each of the following to you when you think about dying?	
Being physically comfortable	
Important	73 (91.3)
Not important	7 (8.8)
Don’t know	0
Being free from pain	
Important	69 (86.3)
Not important	10 (12.5)
Don’t know	1 (1.3)
Saying everything I want to say to people in my family (n = 79)^a^	
Important	77 (97.5)
Not important	2 (2.5)
Don’t know	0
Being at peace spiritually	
Important	74 (92.5)
Not important	4 (5.0)
Don’t know	2 (2.5)
Not being a burden to loved ones	
Important	69 (86.3)
Not important	8 (10.0)
Don’t know	3 (3.8)
Knowing how to say goodbye	
Important	71 (88.8)
Not important	6 (7.5)
Don’t know	3 (3.8)
Having a sense of my own worth or value	
Important	72 (90.0)
Not important	4 (5.0)
Don’t know	4 (5.0)
Being off machines that extend life, such as life support	
Important	48 (60.0)
Not important	27 (33.8)
Don’t know	5 (6.3)
Dying a natural death	
Important	48 (60.0)
Not important	28 (35.0)
Don’t know	4 (5.0)

^a^ Data were missing for 1 adolescent.

The importance of “dying a natural death” and of “being off machines that extend life, if dying” was rated as very important or important by more than one-half of adolescents (48 participants each [60.0%]). Most also thought it would be important to “stay in their own home, if dying” (61 participants [76.2%]). Adolescents were concerned about being a burden to loved ones, ranging from 50% to 86%, depending on how the question was asked.

Exploratory analysis of demographic correlates of adolescent responses with Bonferroni corrections are shown in eTable 2 for age, eTable 3 for sex, eTable 4 for race/ethnicity, and eTable 5 for poverty in the Supplement. Older adolescents (aged ≥18 years) were very comfortable talking about death (12 participants [31.6%] vs 5 participants [11.9%]) and were more likely to have heard of hospice (33 participants [86.8%] vs 23 participants [54.8%]) compared with younger adolescents (aged 14-17 years). Female participants were more likely than male participants to have heard about but not completed an advance directive (22 participants [50.0%] vs 8 participants [22.2%]) and to regard having a sense of their own worth as very important (32 participants [72.3%] vs 17 participants [47.2%]). Nonwhite adolescents were very afraid of dying in an institution (6 participants [31.6%] vs 7 participants [11.7%]) and less likely to have heard of hospice (9 participants [47.4%] vs 46 participants [76.7%]) compared with white adolescents.

### Congruence

**Figure 2.**
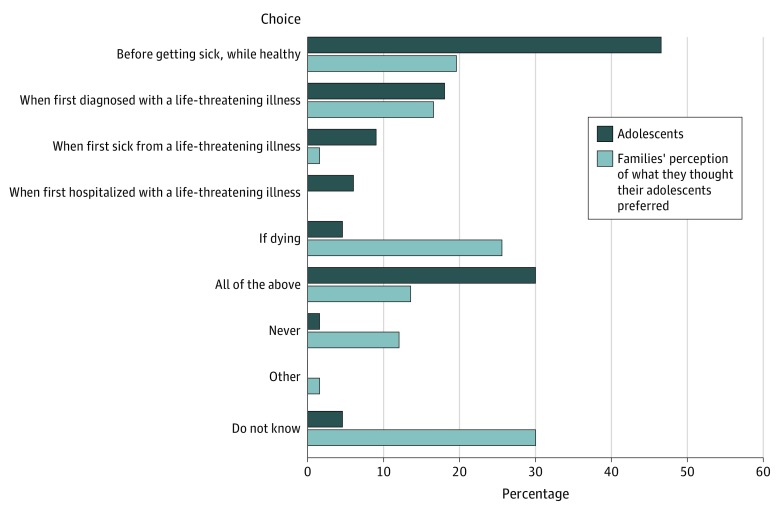
Adolescents’ Self-Report on Best Time for End-of-Life Decisions and Families’ Perception of Their Adolescents’ Beliefs About Best Timing, Among 80 Dyads

Figure 2 illustrates that most adolescents thought the best time to bring up EOL decisions was early, with 86% reporting “before getting sick, while healthy,” “when first diagnosed,” “when first sick from a life-threatening illness,” or “all of the above.” Only 39% of families accurately reported their adolescents’ preference for early timing with poor dyadic congruence (PABAK, 0.18).

Congruence for other responses is reported in Table 3. Excellent congruence was found for the following survey responses: “honest answers from my doctor” (PABAK, 0.95), “understanding my treatment choices” (PABAK, 0.95), “family and friends visiting me” (PABAK, 0.93), and “saying everything I want to say to people in my family” (PABAK, 0.92). There was good congruence on “being at peace spiritually” (PABAK, 0.65). Poor congruence was found on “dying a natural death” (PABAK, 0.18) and “being off machines that extend life, such as life support” (PABAK, 0). eTable 6 in the Supplement shows additional congruence data, including poor congruence for “If someone could tell me when I would die, I would want to know” (PABAK, 0.18).

**Table 3.** Congruence Between Adolescents’ and Families’ Perception of What They Thought Their Adolescents Preferred

Question^a^	Dyads with congruent responses, No. (%) (N = 80)	PABAK^b^
How important would each of the following be to you if you were dealing with your own dying?		
Family and friends visiting me		
Important	77 (96.3)	0.93
Otherwise	0	
Staying in my own home		
Important	53 (66.3)	0.38
Otherwise	2 (2.5)	
Honest answers from my doctor		
Important	78 (97.5)	0.95
Otherwise	0	
Comfort from church services or persons such as a minister, priest, imam, or rabbi		
Important	47 (58.8)	0.58
Otherwise	16 (20.0)	
Planning my own funeral		
Important	29 (36.3)	0.23
Otherwise	20 (25.0)	
Being able to complete an advance directive that would let loved ones know my wishes		
Important	60 (75.0)	0.58
Otherwise	3 (3.8)	
Fulfilling personal goals or pleasure		
Important	72 (90.0)	0.83
Otherwise	1 (1.3)	
Reviewing my life history with my family		
Important	41 (51.3)	0.25
Otherwise	9 (11.3)	
Having health care professionals visit me at my home		
Important	49 (61.3)	0.45
Otherwise	9 (11.3)	
Understanding my treatment choices		
Important	78 (97.5)	0.95
Otherwise	0	
How important are each of the following to you when you think about dying?		
Being physically comfortable		
Important	69 (86.3)	0.73
Otherwise	0	
Being free from pain		
Important	67 (83.8)	0.68
Otherwise	0	
Saying everything I want to say to people in my family (n = 79 dyads)^c^		
Important	75 (94.9)	0.92
Otherwise	1 (1.3)	
Being at peace spiritually		
Important	65 (81.3)	0.65
Otherwise	1 (1.3)	
Not being a burden to loved ones		
Important	56 (70.0)	0.45
Otherwise	2 (2.5)	
Knowing how to say goodbye		
Important	60 (75.0)	0.55
Otherwise	2 (2.5)	
Having a sense of my own worth or value		
Important	62 (77.5)	0.58
Otherwise	1 (1.3)	
Being off machines that extend life, such as life support		
Important	24 (30.0)	0
Otherwise	16 (20.0)	
Dying a natural death		
Important	34 (42.5)	0.18
Otherwise	13 (16.3)	

Abbreviation: PABAK, prevalence-adjusted, bias-adjusted κ.

^a^ Important included very important and somewhat important. Otherwise included neither important nor unimportant, not very important, not at all important, and do not know.

^b^ Less than 0.40 denotes poor, 0.40 to 0.59 denotes fair, 0.60 to 0.74 denotes good, and 0.75 to 1.00 denotes excellent congruence.

^c^ One dyad was excluded because data were missing for the adolescent.

## Discussion

Survey results about congruence in goals, values, and preferences are important for pACP because explication of patient’s goals, values, and preferences helps prepare patients and surrogates to participate with clinicians in making the best possible in-the-moment decisions, as discussed by Sudore and Fried^42^ with adult populations. The decisions themselves are value laden, and without understanding values, congruent decisions may not be made. The survey data support this concern. Despite substantive areas of agreement around the relational aspects of dying, families had a poor understanding of their adolescent’s values with respect to medical interventions, if dying, or the optimal time to have pACP conversations from their child’s perspective. The FACE-TC protocol uses the survey to help initiate conversations, which families identified as important during protocol development. The survey prepares participants for the face-to-face facilitated conversations in session 2 and advance directive completion in session 3. This way there were no surprises for adolescents or families when they returned for sessions 2 and 3 about what we would be talking about. This was and is an important part of our safety plan for human participants’ protections, so we would not unduly distress the patients or their families.

Adolescents with cancer were willing and able to engage in pACP with their families, even though some found the process uncomfortable, and not all would want to be told when they would die. Specific findings should be viewed in light of the 39% participation rate (130 of 333 participants), as discussed later in the Strengths and Limitations section. Of those who declined to participate, 23% (46 of 203 participants) had at least 1 member of the dyad who did not want to discuss pACP, so that the opinions of this subpopulation are not represented (Figure 1).

Most adolescents reported a willingness to forgo life-prolonging medical treatments if dying and to die a natural death. Families’ understanding of these preferences was poor, confirming pilot findings with 17 dyads^8^ and the teams’ HIV study with 48 adolescent-family dyads.^39^ In a medical record review^43^ of patients with cancer aged 15 to 39 years, investigators found that 56% preferred comfort care to life-prolonging care in the month before their death. Yet, 75% of those who preferred comfort measures received at least 1 form of intensive EOL care.^43^ Ongoing communication with families and clinicians regarding adolescents’ preferences is needed.

The optimal timing for initiating conversations about EOL decisions from the adolescents’ perspective is clear and consistent with our pilot cancer study^8^ and adolescent HIV studies.^39,44^ Among adolescents willing to enroll in a trial about pACP, adolescents preferred to talk about EOL decisions when healthy, first diagnosed, or throughout the disease process, rather than when hospitalized or dying. Families consistently thought their child preferred to delay the conversations. Clinically, hospitalization and life-threatening medical crisis are the most frequent triggers for clinicians to initiate EOL conversations, even while recognizing this is often too late.^30,45^

These gaps in understanding have serious clinical consequences because modern medicine has medicalized dying, resulting in most adolescents who die from cancer dying after withholding or withdrawing life-sustaining treatment.^5,6^ These emotional decisions are made by their families, and there is the potential for decisional regret^46^ and long-term complicated grief.^47,48^ The high premium adolescents placed on understanding their treatment choices and saying everything they have to say to their families underlines the importance of timely pACP, which may ease the families’ burden of making EOL decisions on behalf of their child.

The importance of being at peace spiritually at the EOL was almost universal (92.5% of participants), consistent with findings of the cancer pilot study (100% of participants)^8^ and adolescent HIV study (94% of participants).^39^ Spirituality influences the experience of illness, pain, and pediatric EOL decisions.^49,50,51^ Spiritual assessments and appropriate referrals should be provided to ensure culturally sensitive care.^51^

Consistent with study findings, many adolescents with life-limiting conditions perceive themselves as a burden,^52,53,54,55^ which may not reflect how families feel.^52,53^ Some patients base treatment decisions on the perceived burden the treatment creates.^52^ Resolving adolescent feelings regarding being a burden is a need that could be addressed through pACP.

### Strengths and Limitations

To our knowledge, this is the largest dyadic EOL survey of adolescents with cancer and their families. The completion of the surveys in 4 tertiary hospital-based settings increases validity and the likelihood of findings influencing clinical practice. Validity is further enhanced by replication of earlier studies. The overall study achieved the enrollment goal of 130 adolescent-family dyads. Generalizability is increased by the geographically and economically diverse sample.

Limitations include a low participation rate (39%), which affects generalizability. Participation was below the 50% benchmark achieved in our pilot^8^ and HIV trial.^39^ Among those who enrolled, only 28% wanted to know if someone could tell them when they would die, in contrast to 50% in the cancer pilot^9^ and 46% in the HIV study.^39^ Overwhelmingly, adolescents wanted honest answers from their physician, suggesting that many adolescents may only want to know their prognosis, if they ask. Nevertheless, all were willing to participate in a pACP trial, which enabled a discussion of the hypothetical scenarios with their family, regardless of prognosis. These findings highlight the complexity of the pACP process.

Social desirability bias could have occurred with face-to-face administration. We chose this approach to enable monitoring of emotional reactions and to control for issues of literacy, impaired vision, item comprehension, and survey completion. Male adolescents with cancer were statistically significantly more likely to decline participation than female adolescents, although 45.0% of enrolled adolescents were male. Among adults, male patients are less likely to participate in ACP than female patients.^56^ The sample included all adolescents receiving oncology care, reflecting recommendations that ACP occur at all stages for anyone with a serious illness.^57^

## Conclusions

Families had a poor understanding of their adolescent’s values regarding their own EOL care with respect to when to initiate EOL conversations and preference for being off machines that extend life, if dying. Pediatric ACP could minimize these misunderstandings, potentially affecting the broader domain of clinical practice guidelines for quality palliative care.^58^ Access to pACP to increase congruence for interested and ready adolescent-family dyads may be more beneficial than simply asking adolescents about their EOL treatment preferences by helping families with the burdens of making EOL decisions, ensuring that adolescents’ preferences are heard, and opening up conversations on topics that both the adolescent and family member may be thinking about, but avoiding. Ultimately, what is at stake here is excessive and unwanted treatment, leading to unnecessary and avoidable suffering.

## Notes

Supplement. **eTable 1.** Comparison of Patients Enrolled in Study Versus Declined Participation in Study **eTable 2.** Frequencies of Adolescent Responses for the Survey by Age Group at Session 1 (N = 80) **eTable 3.** Frequencies of Adolescent Responses for Survey by Gender at Session 1 (N = 80) **eTable 4.** Frequencies of Adolescent Responses for Survey by Race at Session 1 (N = 79) **eTable 5.** Frequencies of Adolescent Responses for Survey by Poverty at Session 1 (N = 78) **eTable 6.** The Congruence on End of Life Needs for Adolescents Living With Cancer Between Adolescents’ and Families’ Perception of What They Thought Their Adolescent Preferred (N = 80 Dyads)
