# Supplementary material for: Congruence Gaps Between Adolescents With Cancer and Their Families Regarding Values, Goals, and Beliefs About End-of-Life Care
Source: JAMA Netw Open. 2020 May 19;3(5):e205424. doi: 10.1001/jamanetworkopen.2020.5424 (PMC7237980; doi:10.1001/jamanetworkopen.2020.5424)
Supplement: Supplement. — eTable 1. Comparison of Patients Enrolled in Study Versus Declined Participation in Study eTable 2. Frequencies of Adolescent Responses for the Survey by Age Group at Session 1 (N = 80) eTable 3. Frequencies of Adolescent Responses for Survey by Gender at Session 1 (N = 80) eTable 4. Frequencies of Adolescent Responses for Survey by Race at Session 1 (N = 79) eTable 5. Frequencies of Adolescent Responses for Survey by Poverty at Session 1 (N = 78) eTable 6. The Congruence on End of Life Needs for Adolescents Living With Cancer Between Adolescents’ and Families’ Perception of What They Thought Their Adolescent Preferred (N = 80 Dyads) [file jamanetwopen-3-e205424-s001.pdf]

Supplementary Online Content

Friebert S, Grossoehme DH, Baker JN, et al. Congruence gaps between adolescents with cancer and their families regarding values, goals, and beliefs about end-of-life care. *JAMA Netw Open*. 2020;3(5):e205424. doi:10.1001/jamanetworkopen.2020.5424

eTable 1. Comparison of Patients Enrolled in Study Versus Declined Participation in Study

eTable 2. Frequencies of Adolescent Responses for the Survey by Age Group at Session 1 (N=80)

eTable 3. Frequencies of Adolescent Responses for Survey by Gender at Session 1 (N=80)

eTable 4. Frequencies of Adolescent Responses for Survey by Race at Session 1 (N=79)

eTable 5. Frequencies of Adolescent Responses for Survey by Poverty at Session 1 (N=78)

eTable 6. The Congruence on End of Life Needs for Adolescents Living With Cancer Between Adolescents' and Families' Perception of What They Thought Their Adolescent Preferred (N=80 Dyads)

This supplementary material has been provided by the authors to give readers additional information about their work.

© 2020 Friebert S et al. *JAMA Network Open.*

eTable 1. Comparison of Participants Enrolled in Study Versus Declined Participation in Study			
**Variable**	**Eligible/ Participating (N=130 dyads)**	**Eligible/ Declining to participating (N=203 dyads)**	**P-value[a]**
Adolescent Age		N=195	0.165
Mean (SD)	17.0 (1.8)	16.7 (1.9)	
Surrogate Age		N=103	0.994
Mean (SD)	46.0 (8.2)	46.0 (7.4)	
	N (%)	N (%)	
Adolescent Gender		N=198	0.015
Female	73 (56.2)	82 (41.4)	
Male	57 (43.8)	115 (58.1)	
Declined	0 (0.0)	1 (0.5)	
Family Gender		N=182	0.488
Female	107 (82.3)	153 (84.1)	
Male	23 (17.7)	27 (14.8)	
Declined	0 (0.0)	2 (1.1)	
Adolescent Race		N=192	0.183
American Indian or Alaska Native	0 (0.0)	1 (0.5)	
Asian	3 (2.3)	4 (2.1)	
Black or African American	19 (14.6)	20 (10.4)	
White	102 (78.5)	158 (82.3)	
More than one race	5 (3.8)	2 (1.0)	
Declined	1 (0.8)	7 (3.6)	
Family Race		N=172	0.006
American Indian or Alaska Native	1 (0.8)	1 (0.6)	
Asian	3 (2.3)	2 (1.2)	
Black or African American	16 (12.3)	15 (8.7)	
White	105 (80.8)	147 (85.5)	
More than one race	5 (3.8)	0 (0.0)	
Declined	0 (0.0)	7 (4.1)	
Adolescent Ethnicity		N=191	0.885
Hispanic or Latino	5 (3.8)	6 (3.1)	
Not Hispanic or Latino	120 (92.3)	176 (92.1)	
Declined	5 (3.8)	9 (4.7)	
Family Ethnicity		N=173	0.005
Hispanic or Latino	4 (3.1)	2 (1.2)	
Not Hispanic or Latino	125 (96.2)	158 (91.3)	
Declined	1 (0.8)	13 (7.5)	
Diagnosis	N=126	N=191	0.410
Leukemia	42 (33.3)	51 (26.7)	
Lymphoma	19 (15.1)	39 (20.4)	
Solid Tumors	34 (27.0)	47 (24.6)	
Brain Tumor	25 (19.8)	38 (19.9)	
Other	6 (4.8)	12 (6.3)	
Unknown	0 (0.0)	4 (2.1)	
On Active Treatment	N=126	N=68[b]	0.531
Yes	27 (21.4)	12 (17.6)	
No	99 (78.6)	56 (82.4)	

[a]: Two-sided p-values were reported from t-test, Pearson chi-square test and Fisher's exact test.
[b]: The data were collected partially because data collection on decliners started late on 07-11-2018.

© 2020 Friebert S et al. *JAMA Network Open.*

eTable 2. Frequencies of Adolescent Responses for Survey by Age Group at Session 1 (N=80)				
**Question**	**Age 14-17 years (N=42)** N (%)	**Age 18+ years (N=38)** N (%)	**All Ages Combined (N=80)** N (%)	**P-value[a]**
1. Have you ever written down any thoughts about your future health plans?				0.901
Yes, definitely	1 (2.4)	2 (5.3)	3 (3.8)	
Very probably	1 (2.4)	0 (0.0)	1 (1.3)	
Probably	2 (4.8)	3 (7.9)	5 (6.3)	
Probably not	7 (16.7)	4 (10.5)	11 (13.8)	
Definitely no	29 (69.0)	27 (71.1)	56 (70.0)	
Do not know	2 (4.8)	2 (5.3)	4 (5.0)	
2a. Have you ever heard about and completed a Health Care Power of Attorney (HCPA), in which you name someone to make decisions about your health care in case you could not?				0.033
Have heard about and completed	0 (0.0)	2 (5.3)	2 (2.5)	
Have heard about but not completed	14 (33.3)	22 (57.9)	36 (45.0)	
Have not heard about	23 (54.8)	12 (31.6)	35 (43.8)	
Do not know	5 (11.9)	2 (5.3)	7 (8.8)	
2b. Have you ever heard about and completed an Advance Directive or living will, such as the Five Wishes?				0.115
Have heard about and completed	1 (2.4)	1 (2.6)	2 (2.5)	
Have heard about but not completed	11 (26.2)	19 (50.0)	30 (37.5)	
Have not heard about	28 (66.7)	16 (42.1)	44 (55.0)	
Do not know	2 (4.8)	2 (5.3)	4 (5.0)	
3a. Whether you have completed any advance directives/pre-plans or not, have you talked about your wishes for care at the end of life with anyone? Select all that apply.[b]				
Spouse/partner	0 (0.0)	0 (0.0)	0 (0.0)	-
Parents	13 (31.0)	12 (31.6)	25 (31.3)	0.952
Siblings (brother/sister)	2 (4.8)	7 (18.4)	9 (11.3)	0.078
Friends	3 (7.1)	6 (15.8)	9 (11.3)	0.296
Boyfriend/girlfriend	0 (0.0)	4 (10.5)	4 (5.0)	0.047
Lawyer	0 (0.0)	0 (0.0)	0 (0.0)	-
Primary physician	0 (0.0)	1 (2.6)	1 (1.3)	0.475
Clergy (such as minister, rabbi, iman etc.)	0 (0.0)	1 (2.6)	1 (1.3)	0.475
Other	1 (2.4)	0 (0.0)	1 (1.3)	1.000
Have not talked with anyone	28 (66.7)	22 (57.9)	50 (62.5)	0.418
3b. Do you have any children?				
No	42 (100.0)	38 (100.0)	80 (100.0)	-
4. If you were very ill and knew that you would not get better, who would you want to be involved in decisions about your end-of-life care? Select all that apply.[c]				

© 2020 Friebert S et al. *JAMA Network Open.*

eTable 2. (continued) Frequencies of Adolescent Responses for Survey by Age Group at Session 1 (N=80)				
	Age 14-17 years (N=42)	Age 18+ years (N=38)	All Ages Combined (N=80)	P-value[a]
Question	N (%)	N (%)	N (%)	
Myself	31 (73.8)	22 (57.9)	53 (66.3)	0.133
My family	40 (95.2)	37 (97.4)	77 (96.3)	1.000
My doctor	24 (57.1)	22 (57.9)	46 (57.5)	0.946
Someone else	5 (11.9)	3 (7.9)	8 (10.0)	0.715
5. When do you think it is the best time to bring up end-of-life decisions?				0.891
Before getting sick, while healthy	15 (35.7)	16 (42.1)	31 (38.8)	
When first diagnosed with a life-threatening illness	5 (11.9)	7 (18.4)	12 (15.0)	
When first sick from a life-threatening illness	3 (7.1)	3 (7.9)	6 (7.5)	
When first hospitalized with a life-threatening illness	1 (2.4)	0 (0.0)	4 (5.0)	
If dying	2 (4.8)	2 (5.3)	3 (3.8)	
All of the above	1 (2.4)	2 (5.3)	20 (25.0)	
Never	13 (31.0)	7 (18.4)	1 (1.3)	
Other	1 (2.4)	0 (0.0)	1 (1.3)	
Do not know	1 (2.4)	1 (2.6)	2 (2.5)	
6. Who are the best people/best person on the treatment team to bring it up with you? Select all that apply.[d]				
Physician	20 (47.6)	29 (76.3)	49 (61.3)	0.009
Nurse practitioner	15 (35.7)	11 (28.9)	26 (32.5)	0.519
Nurse	14 (33.3)	10 (26.3)	24 (30.0)	0.494
Social worker	9 (21.4)	14 (36.8)	23 (28.8)	0.128
Psychologist	2 (4.8)	11 (28.9)	13 (16.3)	0.005
Case manager	0 (0.0)	5 (13.2)	5 (6.3)	0.021
Chaplain	3 (7.1)	2 (5.3)	5 (6.3)	1
Patient advocate	4 (9.5)	3 (7.9)	7 (8.8)	1
Other	3 (7.1)	1 (2.6)	4 (5.0)	0.617
Do not know	7 (16.7)	4 (10.5)	11 (13.8)	0.525
7. Do you believe that once you make an important medical decision, for example, to be put on a respirator, a machine that breathes for you, that you would be able to change your mind?				0.395
Yes, definitely	13 (31.0)	7 (18.4)	20 (25.0)	
Very probably	5 (11.9)	6 (15.8)	11 (13.8)	
Probably	10 (23.8)	16 (42.1)	26 (32.5)	
Probably not	9 (21.4)	6 (15.8)	15 (18.8)	
Do not know	5 (11.9)	3 (7.9)	8 (10.0)	
8. Do you think your doctor or the hospital will respect your wishes, that is, do what you want about medical care?				0.686
Yes, definitely	23 (54.8)	18 (47.4)	41 (51.3)	
Very probably	11 (26.2)	9 (23.7)	20 (25.0)	

© 2020 Friebert S et al. *JAMA Network Open.*

eTable 2. (continued) Frequencies of Adolescent Responses for Survey by Age Group at Session 1 (N=80)				
	**Age 14-17 years (N=42)**	**Age 18+ years (N=38)**	**All Ages Combined (N=80)**	**P-value[a]**
**Question**	**N (%)**	**N (%)**	**N (%)**	
Probably	4 (9.5)	8 (21.1)	12 (15.0)	
Probably not	2 (4.8)	2 (5.3)	4 (5.0)	
Do not know	2 (4.8)	1 (2.6)	3 (3.8)	
9. Do you think your parent/guardian/surrogate understands your wishes?				0.548
Yes, definitely	24 (58.5)	17 (44.7)	41 (51.9)	
Very probably	7 (17.1)	7 (18.4)	14 (17.7)	
Probably	7 (17.1)	8 (21.1)	15 (19.0)	
Probably not	1 (2.4)	4 (10.5)	5 (6.3)	
Definitely no	1 (2.4)	0 (0.0)	1 (1.3)	
Do not know	1 (2.4)	2 (5.3)	3 (3.8)	
10. Do you think your parent/guardian/surrogate will respect your wishes, that is, do what you want about your medical care?				0.843
Yes, definitely	22 (52.4)	19 (50.0)	41 (51.3)	
Very probably	9 (21.4)	9 (23.7)	18 (22.5)	
Probably	8 (19.0)	6 (15.8)	14 (17.5)	
Probably not	1 (2.4)	3 (7.9)	4 (5.0)	
Do not know	2 (4.8)	1 (2.6)	3 (3.8)	
11. How often has death and dying been talked about in your family?				0.421
Very often	0 (0.0)	2 (5.3)	2 (2.5)	
Often	5 (11.9)	7 (18.4)	12 (15.0)	
Occasionally	13 (31.0)	13 (34.2)	26 (35.5)	
Rarely	19 (45.2)	13 (34.2)	32 (40.0)	
Never	4 (9.5)	1 (2.6)	5 (6.3)	
Do not know	1 (2.4)	2 (5.3)	3 (3.8)	
12. How comfortable are you talking about death?				0.013
Very comfortable	5 (11.9)	12 (31.6)	17 (21.3)	
Somewhat comfortable	15 (35.7)	18 (47.4)	33 (41.3)	
Neither comfortable or uncomfortable	9 (21.4)	7 (18.4)	16 (20.0)	
Not very comfortable	6 (14.3)	1 (2.6)	7 (8.8)	
Not at all comfortable	5 (11.9)	0 (0.0)	5 (6.3)	
Do not know	2 (4.8)	0 (0.0)	2 (2.5)	
13. How likely are you to?				
13a. Attend funerals or memorial services when a loved one, friend or classmate dies				0.847
Very likely	31 (73.8)	30 (78.9)	61 (76.3)	
Somewhat likely	7 (16.7)	5 (13.2)	12 (15.0)	

© 2020 Friebert S et al. *JAMA Network Open.*

eTable 2. (continued) Frequencies of Adolescent Responses for Survey by Age Group at Session 1 (N=80)				
	**Age 14-17 years (N=42)**	**Age 18+ years (N=38)**	**All Ages Combined (N=80)**	**P-value[a]**
**Question**	**N (%)**	**N (%)**	**N (%)**	
Neither likely or unlikely	1 (2.4)	1 (2.6)	2 (2.5)	
Not very likely	0 (0.0)	1 (2.6)	1 (1.3)	
Not at all likely	1 (2.4)	1 (2.6)	2 (2.5)	
Do not know	2 (4.8)	0 (0.0)	2 (2.5)	
13b. Avoid medical checkups because you are afraid the doctor will find "something serious"				0.025
Very likely	2 (4.8)	0 (0.0)	2 (2.5)	
Somewhat likely	5 (11.9)	6 (15.8)	11 (13.8)	
Neither likely or unlikely	1 (2.4)	2 (5.3)	3 (3.8)	
Not very likely	2 (4.8)	10 (26.3)	12 (15.0)	
Not at all likely	31 (73.8)	20 (52.6)	51 (63.8)	
Do not know	1 (2.4)	0 (0.0)	1 (1.3)	
13c. Speak freely to loved ones about death and dying				0.656
Very likely	6 (14.3)	11 (28.9)	17 (21.3)	
Somewhat likely	16 (38.1)	13 (34.2)	29 (36.3)	
Neither likely or unlikely	10 (23.8)	7 (18.4)	17 (21.3)	
Not very likely	4 (9.5)	4 (10.5)	8 (10.0)	
Not at all likely	5 (11.9)	3 (7.9)	8 (10.0)	
Do not know	1 (2.4)	0 (0.0)	1 (1.3)	
13d. Visit or telephone a friend or relative who has recently lost a loved one in order to see how they are doing				1.000
Very likely	30 (71.4)	28 (73.7)	58 (72.5)	
Somewhat likely	9 (21.4)	8 (21.1)	17 (21.3)	
Neither likely or unlikely	2 (4.8)	1 (2.6)	3 (3.8)	
Not very likely	1 (2.4)	1 (2.6)	2 (2.5)	
13e. Preplan your own funeral, for example, choose someone to speak or choose the music you would want				0.350
Very likely	16 (38.1)	10 (26.3)	26 (32.5)	
Somewhat likely	8 (19.0)	7 (18.4)	15 (18.8)	
Neither likely or unlikely	7 (16.7)	6 (15.8)	13 (16.3)	
Not very likely	4 (9.5)	6 (15.8)	10 (12.5)	
Not at all likely	3 (7.1)	8 (21.1)	11 (13.8)	
Do not know	4 (9.5)	1 (2.6)	5 (6.3)	
14. How afraid, if at all, are you of?				
14a. Dying from a long term illness				0.206
Very afraid	7 (16.7)	2 (5.3)	9 (11.3)	
Somewhat afraid	11 (26.2)	14 (36.8)	25 (31.3)	
Neither afraid nor not afraid	3 (7.1)	7 (18.4)	10 (12.5)	

© 2020 Friebert S et al. *JAMA Network Open.*

eTable 2. (continued) Frequencies of Adolescent Responses for Survey by Age Group at Session 1 (N=80)				
Question	Age 14-17 years (N=42) N (%)	Age 18+ years (N=38) N (%)	All Ages Combined (N=80) N (%)	P-value[a]
Not very afraid	8 (19.0)	9 (23.7)	17 (21.3)	
Not at all afraid	11 (26.2)	5 (13.2)	16 (20.0)	
Do not know	2 (4.8)	1 (2.6)	3 (3.8)	
14b. Dying suddenly, such as an accident, or being killed:				0.030
Very afraid	12 (28.6)	2 (5.3)	14 (17.5)	
Somewhat afraid	14 (33.3)	11 (28.9)	25 (31.3)	
Neither afraid nor not afraid	4 (9.5)	10 (26.3)	14 (17.5)	
Not very afraid	6 (14.3)	8 (21.1)	14 (17.5)	
Not at all afraid	5 (11.9)	7 (18.4)	12 (15.0)	
Do not know	1 (2.4)	0 (0.0)	1 (1.3)	
14c. Dying alone				0.420
Very afraid	15 (35.7)	9 (23.7)	24 (30.0)	
Somewhat afraid	5 (11.9)	10 (26.3)	15 (18.8)	
Neither afraid nor not afraid	7 (16.7)	6 (15.8)	13 (16.3)	
Not very afraid	4 (9.5)	6 (15.8)	10 (12.5)	
Not at all afraid	10 (23.8)	7 (18.4)	17 (21.3)	
Do not know	1 (2.4)	0 (0.0)	1 (1.3)	
14d. Dying in an institution such as a nursing home or hospital				0.169
Very afraid	7 (16.7)	6 (15.8)	13 (16.3)	
Somewhat afraid	8 (19.0)	4 (10.5)	12 (15.0)	
Neither afraid nor not afraid	5 (11.9)	10 (26.3)	15 (18.8)	
Not very afraid	6 (14.3)	10 (26.3)	16 (20.0)	
Not at all afraid	13 (31.0)	8 (21.1)	21 (26.3)	
Do not know	3 (7.1)	0 (0.0)	3 (3.8)	
14e. Dying painfully				0.865
Very afraid	15 (35.7)	12 (31.6)	27 (33.8)	
Somewhat afraid	14 (33.3)	12 (31.6)	26 (32.5)	
Neither afraid nor not afraid	4 (9.5)	3 (7.9)	7 (8.8)	
Not very afraid	3 (7.1)	6 (15.8)	9 (11.3)	
Not at all afraid	5 (11.9)	5 (13.2)	10 (12.5)	
Do not know	1 (2.4)	0 (0.0)	1 (1.3)	
15. How strongly do you agree or disagree that?				
15a. Dying is an important part of life				0.181
Strongly agree	13 (31.0)	20 (52.6)	33 (41.3)	
Agree	20 (47.6)	10 (26.3)	30 (37.5)	
Neither agree or disagree	5 (11.9)	4 (10.5)	9 (11.3)	
Disagree	3 (7.1)	3 (7.9)	6 (7.5)	

© 2020 Friebert S et al. *JAMA Network Open.*

eTable 2. (continued) Frequencies of Adolescent Responses for Survey by Age Group at Session 1 (N=80)				
	Age 14-17 years (N=42)	Age 18+ years (N=38)	All Ages Combined (N=80)	P-value[a]
Question	N (%)	N (%)	N (%)	
Strongly disagree	1 (2.4)	0 (0.0)	1 (1.3)	
Do not know	0 (0.0)	1 (2.6)	1 (1.3)	
15b. If someone could tell me when I would die, I would want to know.				0345
Strongly agree	11 (26.2)	5 (13.2)	16 (20.0)	
Agree	9 (21.4)	5 (13.2)	14 (17.5)	
Neither agree or disagree	5 (11.9)	5 (13.2)	10 (12.5)	
Disagree	3 (7.1)	8 (21.1)	11 (13.8)	
Strongly disagree	12 (28.6)	12 (31.6)	24 (30.0)	
Do not know	2 (4.8)	3 (7.9)	5 (6.3)	
16. When you think about death and dying, how concerned are you that?				
16a. The family's money won't last:				0.371
Very concerned	3 (7.1)	0 (0.0)	3 (3.8)	
Concerned	9 (21.4)	11 (28.9)	20 (25.0)	
Neither concerned nor unconcerned	12 (28.6)	9 (23.7)	21 (26.3)	
Not concerned	5 (11.9)	8 (21.1)	13 (16.3)	
Not at all concerned	9 (21.4)	9 (23.7)	18 (22.5)	
Do not know	4 (9.5)	1 (2.6)	5 (6.3)	
16b. I will be a burden to, or overload, my family or friends.				0.050
Very concerned	5 (11.9)	8 (21.1)	13 (16.3)	
Concerned	16 (38.1)	11 (28.9)	27 (33.8)	
Neither concerned nor unconcerned	7 (16.7)	0 (0.0)	7 (8.8)	
Not concerned	7 (16.7)	12 (31.6)	19 (23.8)	
Not at all concerned	6 (14.3)	5 (13.2)	11 (13.8)	
Do not know	1 (2.4)	2 (5.3)	3 (3.8)	
17. Which of the following health problems, if any, do you think are worse than death? Select all that apply.[e]				
Living with great pain	18 (42.9)	15 (39.5)	33 (41.3)	0.759
Total physical dependency on others, for example, being in a wheelchair	11 (26.2)	12 (31.6)	23 (28.8)	0.595
Not being able to communicate my wishes and/or care to family members, for example, being in a coma Checked	22 (52.4)	26 (68.4)	48 (60.0)	0.144
None are worse than death	12 (28.6)	9 (23.7)	21 (26.3)	0.620
18. How important would each of the following be to you if you were dealing with your own dying?				
18a. Family/friends visiting you				0.518
Very important	34 (81.0)	35 (92.1)	69 (86.3)	

© 2020 Friebert S et al. *JAMA Network Open.*

eTable 2. (continued) Frequencies of Adolescent Responses for Survey by Age Group at Session 1 (N=80)				
	**Age 14-17 years (N=42)**	**Age 18+ years (N=38)**	**All Ages Combined (N=80)**	**P-value[a]**
**Question**	**N (%)**	**N (%)**	**N (%)**	
Somewhat important	6 (14.3)	2 (5.3)	8 (10.0)	
Neither important nor unimportant	1 (2.4)	1 (2.6)	2 (2.5)	
Do not know	1 (2.4)	0 (0.0)	1 (1.3)	
18b. Being able to stay in your own home				0.533
Very important	15 (35.7)	12 (31.6)	27 (33.8)	
Somewhat important	16 (38.1)	18 (47.4)	34 (42.5)	
Neither important nor unimportant	5 (11.9)	2 (5.3)	7 (8.8)	
Not very important	5 (11.9)	4 (10.5)	9 (11.3)	
Not at all important	0 (0.0)	2 (5.3)	2 (2.5)	
Do not know	1 (2.4)	0 (0.0)	1 (1.3)	
18c. Honest answers from your doctor				0.602
Very important	41 (97.6)	36 (94.7)	77 (96.3)	
Somewhat important	1 (2.4)	2 (5.3)	3 (3.8)	
18d. Comfort from church services or persons such as a minister, priest, imam, or rabbi				0.701
Very important	18 (42.9)	12 (31.6)	30 (37.5)	
Somewhat important	9 (21.4)	13 (34.2)	22 (27.5)	
Neither important nor unimportant	4 (9.5)	5 (13.2)	9 (11.3)	
Not very important	3 (7.1)	3 (7.9)	6 (7.5)	
Not at all important	7 (16.7)	5 (13.2)	12 (15.0)	
Do not know	1 (2.4)	0 (0.0)	1 (1.3)	
18e. Planning your own funeral				0.109
Very important	11 (26.2)	5 (13.2)	16 (20.0)	
Somewhat important	13 (31.0)	14 (36.8)	27 (33.8)	
Neither important nor unimportant	11 (26.2)	8 (21.1)	19 (23.8)	
Not very important	3 (7.1)	9 (23.7)	12 (15.0)	
Not at all important	1 (2.4)	2 (5.3)	3 (3.8)	
Do not know	3 (7.1)	0 (0.0)	3 (3.8)	
18f. Being able to complete an advance directive that would let loved ones know your wishes, if you were unable to speak for yourself.				0.072
Very important	24 (57.1)	19 (50.0)	43 (53.8)	
Somewhat important	11 (26.2)	17 (44.7)	28 (35.0)	
Neither important nor unimportant	2 (4.8)	2 (5.3)	4 (5.0)	
Do not know	5 (11.9)	0 (0.0)	5 (6.3)	
18g. Fulfilling personal goals/pleasures				0.575
Very important	27 (64.3)	28 (73.7)	55 (68.8)	
Somewhat important	12 (28.6)	10 (26.3)	22 (27.5)	

© 2020 Friebert S et al. *JAMA Network Open.*

eTable 2. (continued) Frequencies of Adolescent Responses for Survey by Age Group at Session 1 (N=80)				
	**Age 14-17 years (N=42)**	**Age 18+ years (N=38)**	**All Ages Combined (N=80)**	**P-value[a]**
**Question**	**N (%)**	**N (%)**	**N (%)**	
Neither important nor unimportant	2 (4.8)	0 (0.0)	2 (2.5)	
Not very important	1 (2.4)	0 (0.0)	1 (1.3)	
18h. Reviewing your life history with your family				0.049
Very important	23 (54.8)	11 (28.9)	34 (42.5)	
Somewhat important	8 (19.0)	15 (39.5)	23 (28.8)	
Neither important nor unimportant	5 (11.9)	9 (23.7)	14 (17.5)	
Not very important	5 (11.9)	3 (7.9)	8 (10.0)	
Do not know	1 (2.4)	0 (0.0)	1 (1.3)	
18i. Having health care professionals visit you at your home				0.737
Very important	12 (28.6)	12 (31.6)	24 (30.0)	
Somewhat important	17 (40.5)	17 (44.7)	34 (42.5)	
Neither important nor unimportant	10 (23.8)	8 (21.1)	18 (22.5)	
Not very important	2 (4.8)	0 (0.0)	2 (2.5)	
Not at all important	0 (0.0)	1 (2.6)	1 (1.3)	
Do not know	1 (2.4)	0 (0.0)	1 (1.3)	
18j. Understanding your treatment choices				0.498
Very important	38 (90.5)	35 (92.1)	73 (91.3)	
Somewhat important	2 (4.8)	3 (7.9)	5 (6.3)	
Do not know	2 (4.8)	0 (0.0)	2 (2.5)	
19. How important are each of the following is to you when you think about dying?				
19a. Being physically comfortable				0.189
Very important	27 (64.3)	22 (57.9)	49 (61.3)	
Somewhat important	13 (31.0)	11 (28.9)	24 (30.0)	
Neither important nor unimportant	0 (0.0)	4 (10.5)	4 (5.0)	
Not very important	1 (2.4)	0 (0.0)	1 (1.3)	
Not at all important	1 (2.4)	1 (2.6)	2 (2.5)	
19b. Being free from pain				0.138
Very important	27 (64.3)	24 (63.2)	51 (63.8)	
Somewhat important	11 (26.2)	7 (18.4)	18 (22.5)	
Neither important nor unimportant	0 (0.0)	5 (13.2)	5 (6.3)	
Not very important	2 (4.8)	1 (2.6)	3 (3.8)	
Not at all important	1 (2.4)	1 (2.6)	2 (2.5)	
Do not know	1 (2.4)	0 (0.0)	1 (1.3)	
19c. Saying everything I want to say to people in my family				0.383
Very important	31 (75.6)	32 (84.2)	63 (79.8)	
Somewhat important	9 (22.0)	5 (13.2)	14 (17.7)	
Neither important nor unimportant	0 (0.0)	1 (2.6)	1 (1.3)	

© 2020 Friebert S et al. *JAMA Network Open.*

eTable 2. (continued) Frequencies of Adolescent Responses for Survey by Age Group at Session 1 (N=80)				
	**Age 14-17 years (N=42)**	**Age 18+ years (N=38)**	**All Ages Combined (N=80)**	**P-value[a]**
**Question**	**N (%)**	**N (%)**	**N (%)**	
Not at all important	1 (2.4)	0 (0.0)	1 (1.3)	
19d. Being at peace spiritually				0.761
Very important	29 (69.0)	30 (78.9)	59 (73.8)	
Somewhat important	9 (21.4)	6 (15.8)	15 (18.8)	
Neither important nor unimportant	1 (2.4)	1 (2.6)	2 (2.5)	
Not very important	1 (2.4)	1 (2.6)	2 (2.5)	
Do not know	2 (4.8)	0 (0.0)	2 (2.5)	
19e. Not being a burden to loved ones				0.988
Very important	28 (66.7)	26 (68.4)	54 (67.5)	
Somewhat important	8 (19.0)	7 (18.4)	15 (18.8)	
Neither important nor unimportant	3 (7.1)	2 (5.3)	5 (6.3)	
Not very important	1 (2.4)	2 (5.3)	3 (3.8)	
Do not know	2 (4.8)	1 (2.6)	3 (3.8)	
19f. Knowing how to say good bye				1.000
Very important	31 (73.8)	28 (73.7)	59 (73.8)	
Somewhat important	6 (14.3)	6 (15.8)	12 (15.0)	
Neither important nor unimportant	2 (4.8)	2 (5.3)	4 (5.0)	
Not very important	1 (2.4)	1 (2.6)	2 (2.5)	
Do not know	2 (4.8)	1 (2.6)	3 (3.8)	
19g. Having a sense of my own worth or value				0.086
Very important	30 (71.4)	19 (50.0)	49 (61.3)	
Somewhat important	8 (19.0)	15 (39.5)	23 (28.8)	
Neither important nor unimportant	1 (2.4)	3 (7.9)	4 (5.0)	
Do not know	3 (7.1)	1 (2.6)	4 (5.0)	
19h. Being off machines that extend life, such as life support.				0.879
Very important	13 (31.0)	10 (26.3)	23 (28.8)	
Somewhat important	15 (35.7)	10 (26.3)	25 (31.3)	
Neither important nor unimportant	9 (21.4)	12 (31.6)	21 (26.3)	
Not very important	2 (4.8)	2 (5.3)	4 (5.0)	
Not at all important	1 (2.4)	1 (2.6)	2 (2.5)	
Do not know	2 (4.8)	3 (7.9)	5 (6.3)	
19i. Dying a natural death				0.230
Very important	16 (38.1)	9 (23.7)	25 (31.3)	
Somewhat important	12 (28.6)	11 (28.9)	23 (28.8)	
Neither important nor unimportant	9 (21.4)	11 (28.9)	20 (25.0)	
Not very important	4 (9.5)	2 (5.3)	6 (7.5)	
Not at all important	1 (2.4)	1 (2.6)	2 (2.5)	

© 2020 Friebert S et al. *JAMA Network Open.*

eTable 2. (continued) Frequencies of Adolescent Responses for Survey by Age Group at Session 1 (N=80)				
	Age 14-17 years (N=42)	Age 18+ years (N=38)	All Ages Combined (N=80)	P-value[a]
Question	N (%)	N (%)	N (%)	
Do not know	0 (0.0)	4 (10.5)	4 (5.0)	
20. If death were likely to happen in the next few weeks, and you could choose where to die, where would you MOST want to die?				0.743
At home without hospice	13 (31.0)	10 (26.3)	23 (28.8)	
At home in hospice	9 (21.4)	11 (28.9)	20 (25.0)	
In a hospital	7 (16.7)	3 (7.9)	10 (12.5)	
No preference	6 (14.3)	6 (15.8)	12 (15.0)	
Do not know	7 (16.7)	8 (21.1)	15 (18.8)	
21. Below are some statements related to pain near the end-of-life that have been expressed by people. How strongly do you agree or disagree with each statement?				
21a. I am afraid the doctor may not believe I am in pain and treat my pain				0.127
Strongly agree	1 (2.4)	1 (2.6)	2 (2.5)	
Agree	8 (19.0)	5 (13.2)	13 (16.3)	
Neither agree or disagree	2 (4.8)	8 (21.1)	10 (12.5)	
Disagree	14 (33.3)	16 (42.1)	30 (37.5)	
Strongly disagree	12 (28.6)	7 (18.4)	19 (23.8)	
Do not know	5 (11.9)	1 (2.6)	6 (7.5)	
21b. I would only take pain medicines when the pain is severe				0.051
Strongly agree	16 (38.1)	9 (23.7)	25 (31.3)	
Agree	15 (35.7)	13 (34.2)	28 (35.0)	
Neither agree or disagree	6 (14.3)	4 (10.5)	10 (12.5)	
Disagree	3 (7.1)	12 (31.6)	15 (18.8)	
Strongly disagree	1 (2.4)	0 (0.0)	1 (1.3)	
Do not know	1 (2.4)	0 (0.0)	1 (1.3)	
21c. I am afraid I will become addicted to the pain medicines over time				0.214
Strongly agree	0 (0.0)	3 (7.9)	3 (3.8)	
Agree	8 (19.0)	9 (23.7)	17 (21.3)	
Neither agree or disagree	1 (2.4)	4 (10.5)	5 (6.3)	
Disagree	15 (35.7)	9 (23.7)	24 (30.0)	
Strongly disagree	15 (35.7)	12 (31.6)	27 (33.8)	
Do not know	3 (7.1)	1 (2.6)	4 (5.0)	
21d. I would take the lowest amount of medicine possible to save larger doses for later when the pain is worse				0.543
Strongly agree	10 (23.8)	5 (13.2)	15 (18.8)	
Agree	17 (40.5)	14 (36.8)	31 (38.8)	
Neither agree or disagree	6 (14.3)	6 (15.8)	12 (15.0)	

© 2020 Friebert S et al. *JAMA Network Open.*

eTable 2. (continued) Frequencies of Adolescent Responses for Survey by Age Group at Session 1 (N=80)				
	Age 14-17 years (N=42)	Age 18+ years (N=38)	All Ages Combined (N=80)	P-value[a]
Question	N (%)	N (%)	N (%)	
Disagree	5 (11.9)	6 (15.8)	11 (13.8)	
Strongly disagree	2 (4.8)	6 (15.8)	8 (10.0)	
Do not know	2 (4.8)	1 (2.6)	3 (3.8)	
21e. I am afraid I would be given too much pain medicine				0.378
Strongly agree	1 (2.4)	5 (13.2)	6 (7.5)	
Agree	10 (23.8)	4 (10.5)	14 (17.5)	
Neither agree or disagree	5 (11.9)	5 (13.2)	10 (12.5)	
Disagree	15 (35.7)	14 (36.8)	29 (36.3)	
Strongly disagree	9 (21.4)	9 (23.7)	18 (22.5)	
Do not know	2 (4.8)	1 (2.6)	3 (3.8)	
22. Have you heard of hospice services?				0.003
Yes	23 (54.8)	33 (86.8)	56 (70.0)	
No	19 (45.2)	5 (13.2)	24 (30.0)	
23. How did you learn about hospice services? (N=56)				0.295
I know someone who used hospice services	9 (39.1)	20 (60.6)	29 (51.8)	
I heard from a health care professional	3 (13.0)	1 (3.0)	4 (7.1)	
I read literature/newspaper/TV/radio/other media	3 (13.0)	4 (12.1)	7 (12.5)	
I heard from others	8 (34.8)	8 (24.2)	16 (28.6)	
24. If you were dying, would you want hospice support? (N=56)				1.000
Yes	10 (43.5)	14 (42.4)	24 (42.9)	
No	2 (8.7)	4 (12.1)	6 (10.7)	
Don't know/not sure	11 (47.8)	15 (45.5)	26 (46.4)	
25. Do you consider yourself religious/spiritual?				0.129
Very religious/spiritual	11 (26.2)	10 (26.3)	21 (26.3)	
Somewhat religious/spiritual	16 (38.1)	23 (60.5)	39 (48.8)	
Not very religious/spiritual	6 (14.3)	3 (7.9)	9 (11.3)	
Not religious/spiritual	8 (19.0)	2 (5.3)	10 (12.5)	
Declined	1 (2.4)	0 (0.0)	1 (1.3)	
26. How often do you attend religious or spiritual services? (N=69)				0.504
Always	4 (12.1)	2 (5.6)	6 (8.7)	
Very often	8 (24.2)	8 (22.2)	16 (23.2)	
Sometimes	16 (48.5)	16 (44.4)	32 (46.4)	
Rarely	5 (15.2)	7 (19.4)	12 (17.4)	
Never	0 (0.0)	3 (8.3)	3 (4.4)	
27. How often does religion or spirituality help you face your fears or do what you were afraid to do? (N=69)				0.670
Everyday	11 (33.3)	7 (19.4)	18 (26.1)	

© 2020 Friebert S et al. *JAMA Network Open.*

eTable 2. (continued) Frequencies of Adolescent Responses for Survey by Age Group at Session 1 (N=80)				
	**Age 14-17 years (N=42)**	**Age 18+ years (N=38)**	**All Ages Combined (N=80)**	**P-value[a]**
**Question**	**N (%)**	**N (%)**	**N (%)**	
A few times a week	8 (24.2)	9 (25.0)	17 (24.6)	
A few times a month	8 (24.2)	10 (27.8)	18 (26.1)	
Rarely	4 (12.1)	8 (22.2)	12 (17.4)	
Never	2 (6.1)	2 (5.6)	4 (5.8)	
30. HOW MANY NIGHTS did you stay in a hospital?				0.498
0	38 (90.5)	33 (86.8)	71 (88.8)	
1-2	1 (2.4)	0 (0.0)	1 (1.3)	
3-5	1 (2.4)	3 (7.9)	4 (5.0)	
6-10	2 (4.8)	1 (2.6)	3 (3.8)	
>20	0 (0.0)	1 (2.6)	1 (1.3)	
31. How healthy are you feeling right now?				0.453
Excellent health	13 (31.0)	9 (23.7)	22 (27.5)	
Very good health	18 (42.9)	13 (34.2)	31 (38.8)	
Good health	6 (14.3)	9 (23.7)	15 (18.8)	
Fair health	4 (9.5)	5 (13.2)	9 (11.3)	
Poor health	0 (0.0)	2 (5.3)	2 (2.5)	
Declined	1 (2.4)	0 (0.0)	1 (1.3)	
32a. Is your mother alive?				1.000
Yes	41 (97.6)	38 (100.0)	79 (98.8)	
No	1 (2.4)	0 (0.0)	1 (1.3)	
32b. Is your father alive?				
Yes	37 (88.1)	36 (94.7)	73 (91.3)	0.678
No	3 (7.1)	2 (5.3)	5 (6.3)	
Do not know	2 (4.8)	0 (0.0)	2 (2.5)	

[a]: Pearson chi-square test or Fisher' exact test.
[b]: Bonferroni corrected significant level is 0.05/8=0.006.
[c]: Bonferroni corrected significant level is 0.05/4=0.013
[d]: Bonferroni corrected significant level is 0.05/10=0.005.
[e]: Bonferroni corrected significant level is 0.05/4=0.013.

© 2020 Friebert S et al. *JAMA Network Open.*

eTable 3. Frequencies of Adolescent Responses for Survey by Gender at Session 1 (N=80)			
	Female (N=44)	Male (N=36)	P-value[a]
Question	N (%)	N (%)	
1. Have you ever written down any thoughts about your future health plans?			0.334
Yes, definitely	3 (6.8)	0 (0.0)	
Very probably	0 (0.0)	1 (2.8)	
Probably	4 (9.1)	1 (2.8)	
Probably not	6 (13.6)	5 (13.9)	
Definitely no	28 (63.6)	28 (77.8)	
Do not know	3 (6.8)	1 (2.8)	
2a. Have you ever heard about and completed a Health Care Power of Attorney (HCPA), in which you name someone to make decisions about your health care in case you could not?			0.069
Have heard about and completed	2 (4.5)	0 (0.0)	
Have heard about but not completed	24 (54.5)	12 (33.3)	
Have not heard about	16 (36.4)	19 (52.8)	
Do not know	2 (4.5)	5 (13.9)	
2b. Have you ever heard about and completed an Advance Directive or living will, such as the Five Wishes?			0.007
Have heard about and completed	1 (2.3)	1 (2.8)	
Have heard about but not completed	22 (50.0)	8 (22.2)	
Have not heard about	21 (47.7)	23 (63.9)	
Do not know	0 (0.0)	4 (11.1)	
3a. Whether you have completed any advance directives/pre-plans or not, have you talked about your wishes for care at the end of life with anyone? Select all that apply.[b]			
Spouse/partner	0 (0.0)	0 (0.0)	-
Parents	12 (27.3)	13 (36.1)	0.396
Siblings (brother/sister)	4 (9.1)	5 (13.9)	0.724
Friends	6 (13.6)	3 (8.3)	0.504
Boyfriend/girlfriend	3 (6.8)	1 (2.8)	0.623
Lawyer	0 (0.0)	0 (0.0)	-
Primary physician	1 (2.3)	0 (0.0)	1.000
Clergy (such as minister, rabbi, iman etc.)	1 (2.3)	0 (0.0)	1.000
Other	0 (0.0)	1 (2.8)	0.45
Have not talked with anyone	31 (70.5)	19 (52.8)	0.104
3b. Do you have any children?			-
No	44 (100.0)	36 (100.0)	
4. If you were very ill and knew that you would not get better, who would you want to be involved in decisions about your end-of-life care? Select all that apply.[c]			

© 2020 Friebert S et al. *JAMA Network Open.*

**eTable 3. (continued) Frequencies of Adolescent Responses for Survey by Gender at Session 1 (N=80)**			
**Question**	**Female (N=44)** N (%)	**Male (N=36)** N (%)	**P-value[a]**
Myself	30 (68.2)	23 (63.9)	0.686
My family	42 (95.5)	35 (97.2)	1.000
My doctor	26 (59.1)	20 (55.6)	0.750
Someone else	5 (11.4)	3 (8.3)	0.724
5. When do you think it is the best time to bring up end-of-life decisions?			0.352
Before getting sick, while healthy	18 (40.9)	13 (36.1)	
When first diagnosed with a life-threatening illness	4 (9.1)	8 (22.2)	
When first sick from a life-threatening illness	4 (9.1)	2 (5.6)	
When first hospitalized with a life-threatening illness	0 (0.0)	1 (2.8)	
If dying	4 (9.1)	0 (0.0)	
All of the above	1 (2.3)	2 (5.6)	
Never	11 (25.0)	9 (25.0)	
Other	1 (2.3)	0 (0.0)	
Do not know	1 (2.3)	1 (2.8)	
6. Who are the best people/best person on the treatment team to bring it up with you? Select all that apply.[d]			
Physician	28 (63.6)	21 (58.3)	0.628
Nurse practitioner	14 (31.8)	12 (33.3)	0.886
Nurse	16 (36.4)	8 (22.2)	0.170
Social worker	16 (36.4)	7 (19.4)	0.096
Psychologist	7 (15.9)	6 (16.7)	0.927
Case manager	3 (6.8)	2 (5.6)	1.000
Chaplain	2 (4.5)	3 (8.3)	0.653
Patient advocate	5 (11.4)	2 (5.6)	0.449
Other	1 (2.3)	3 (8.3)	0.322
Do not know	7 (15.9)	4 (11.1)	0.746
7. Do you believe that once you make an important medical decision, for example, to be put on a respirator, a machine that breathes for you, that you would be able to change your mind?			0.079
Yes, definitely	9 (20.5)	11 (30.6)	
Very probably	10 (22.7)	1 (2.8)	
Probably	15 (34.1)	11 (30.6)	
Probably not	7 (15.9)	8 (22.2)	
Do not know	3 (6.8)	5 (13.9)	
8. Do you think your doctor or the hospital will respect your wishes, that is, do what you want about medical care?			0.102
Yes, definitely	27 (61.4)	14 (38.9)	
Very probably	8 (18.2)	12 (33.3)	

© 2020 Friebert S et al. *JAMA Network Open.*

**eTable 3. (continued) Frequencies of Adolescent Responses for Survey by Gender at Session 1 (N=80)**			
**Question**	**Female (N=44)** **N (%)**	**Male (N=36)** **N (%)**	**P-value[a]**
Probably	7 (15.9)	5 (13.9)	
Probably not	2 (4.5)	2 (5.6)	
Do not know	0 (0.0)	3 (8.3)	
9. Do you think your parent/guardian/surrogate understands your wishes?			0.919
Yes, definitely	22 (51.2)	19 (52.8)	
Very probably	8 (18.6)	6 (16.7)	
Probably	9 (20.9)	6 (16.7)	
Probably not	2 (4.7)	3 (8.3)	
Definitely no	1 (2.3)	0 (0.0)	
Do not know	1 (2.3)	2 (5.6)	
10. Do you think your parent/guardian/surrogate will respect your wishes, that is, do what you want about your medical care?			0.156
Yes, definitely	27 (61.4)	14 (38.9)	
Very probably	8 (18.2)	10 (27.8)	
Probably	7 (15.9)	7 (19.4)	
Probably not	2 (4.5)	2 (5.6)	
Do not know	0 (0.0)	3 (8.3)	
11. How often has death and dying been talked about in your family?			0.301
Very often	0 (0.0)	2 (5.6)	
Often	5 (11.4)	7 (19.4)	
Occasionally	18 (40.9)	8 (22.2)	
Rarely	17 (38.6)	15 (41.7)	
Never	3 (6.8)	2 (5.6)	
Do not know	1 (2.3)	2 (5.6)	
12. How comfortable are you talking about death?			0.178
Very comfortable	10 (22.7)	7 (19.4)	
Somewhat comfortable	19 (43.2)	14 (38.9)	
Neither comfortable or uncomfortable	8 (18.2)	8 (22.2)	
Not very comfortable	1 (2.3)	6 (16.7)	
Not at all comfortable	4 (9.1)	1 (2.8)	
Do not know	2 (4.5)	0 (0.0)	
13. How likely are you to?			
13a. Attend funerals or memorial services when a loved one, friend or classmate dies			0.558
Very likely	34 (77.3)	27 (75.0)	
Somewhat likely	8 (18.2)	4 (11.1)	
Neither likely or unlikely	0 (0.0)	2 (5.6)	

© 2020 Friebert S et al. *JAMA Network Open.*

eTable 3. (continued) Frequencies of Adolescent Responses for Survey by Gender at Session 1 (N=80)			
	**Female (N=44)**	**Male (N=36)**	**P-value[a]**
**Question**	**N (%)**	**N (%)**	
Not very likely	0 (0.0)	1 (2.8)	
Not at all likely	1 (2.3)	1 (2.8)	
Do not know	1 (2.3)	1 (2.8)	
13b. Avoid medical checkups because you are afraid the doctor will find "something serious"			0.662
Very likely	1 (2.3)	1 (2.8)	
Somewhat likely	6 (13.6)	5 (13.9)	
Neither likely or unlikely	1 (2.3)	2 (5.6)	
Not very likely	5 (11.4)	7 (19.4)	
Not at all likely	31 (70.5)	20 (55.6)	
Do not know	0 (0.0)	1 (2.8)	
13c. Speak freely to loved ones about death and dying			0.977
Very likely	9 (20.5)	8 (22.2)	
Somewhat likely	16 (36.4)	13 (36.1)	
Neither likely or unlikely	8 (18.2)	9 (25.0)	
Not very likely	5 (11.4)	3 (8.3)	
Not at all likely	5 (11.4)	3 (8.3)	
Do not know	1 (2.3)	0 (0.0)	
13d. Visit or telephone a friend or relative who has recently lost a loved one in order to see how they are doing			0.877
Very likely	33 (75.0)	25 (69.4)	
Somewhat likely	9 (20.5)	8 (22.2)	
Neither likely or unlikely	1 (2.3)	2 (5.6)	
Not very likely	1 (2.3)	1 (2.8)	
13e. Preplan your own funeral, for example, choose someone to speak or choose the music you would want			0.799
Very likely	14 (31.8)	12 (33.3)	
Somewhat likely	10 (22.7)	5 (13.9)	
Neither likely or unlikely	8 (18.2)	5 (13.9)	
Not very likely	4 (9.1)	6 (16.7)	
Not at all likely	6 (13.6)	5 (13.9)	
Do not know	2 (4.5)	3 (8.3)	
14. How afraid, if at all, are you of?			
14a. Dying from a long term illness			0.359
Very afraid	7 (15.9)	2 (5.6)	
Somewhat afraid	11 (25.0)	14 (38.9)	
Neither afraid nor not afraid	4 (9.1)	6 (16.7)	
Not very afraid	11 (25.0)	6 (16.7)	
Not at all afraid	10 (22.7)	6 (16.7)	

© 2020 Friebert S et al. *JAMA Network Open.*

eTable 3. (continued) Frequencies of Adolescent Responses for Survey by Gender at Session 1 (N=80)			
Question	Female (N=44) N (%)	Male (N=36) N (%)	P-value[a]
Do not know	1 (2.3)	2 (5.6)	
14b. Dying suddenly, such as an accident, or being killed:			0.794
Very afraid	6 (13.6)	8 (22.2)	
Somewhat afraid	16 (36.4)	9 (25.0)	
Neither afraid nor not afraid	7 (15.9)	7 (19.4)	
Not very afraid	8 (18.2)	6 (16.7)	
Not at all afraid	6 (13.6)	6 (16.7)	
Do not know	1 (2.3)	0 (0.0)	
14c. Dying alone			0.922
Very afraid	13 (29.5)	11 (30.6)	
Somewhat afraid	9 (20.5)	6 (16.7)	
Neither afraid nor not afraid	6 (13.6)	7 (19.4)	
Not very afraid	6 (13.6)	4 (11.1)	
Not at all afraid	10 (22.7)	7 (19.4)	
Do not know	0 (0.0)	1 (2.8)	
14d. Dying in an institution such as a nursing home or hospital			0.911
Very afraid	7 (15.9)	6 (16.7)	
Somewhat afraid	6 (13.6)	6 (16.7)	
Neither afraid nor not afraid	10 (22.7)	5 (13.9)	
Not very afraid	9 (20.5)	7 (19.4)	
Not at all afraid	10 (22.7)	11 (30.6)	
Do not know	2 (4.5)	1 (2.8)	
14e. Dying painfully			0.194
Very afraid	12 (27.3)	15 (41.7)	
Somewhat afraid	18 (40.9)	8 (22.2)	
Neither afraid nor not afraid	3 (6.8)	4 (11.1)	
Not very afraid	3 (6.8)	6 (16.7)	
Not at all afraid	7 (15.9)	3 (8.3)	
Do not know	1 (2.3)	0 (0.0)	
15. How strongly do you agree or disagree that?			
15a. Dying is an important part of life			0.145
Strongly agree	18 (40.9)	15 (41.7)	
Agree	20 (45.5)	10 (27.8)	
Neither agree or disagree	4 (9.1)	5 (13.9)	
Disagree	1 (2.3)	5 (13.9)	
Strongly disagree	0 (0.0)	1 (2.8)	
Do not know	1 (2.3)	0 (0.0)	

© 2020 Friebert S et al. *JAMA Network Open.*

Question	Female (N=44) N (%)	Male (N=36) N (%)	P-value[a]
15b. If someone could tell me when I would die, I would want to know.			0.406

eTable 3. (continued) Frequencies of Adolescent Responses for Survey by Gender at Session 1 (N=80)

Question	Female (N=44) N (%)	Male (N=36) N (%)	P-value[a]
Strongly agree	7 (15.9)	9 (25.0)	
Agree	8 (18.2)	6 (16.7)	
Neither agree or disagree	8 (18.2)	2 (5.6)	
Disagree	5 (11.4)	6 (16.7)	
Strongly disagree	12 (27.3)	12 (33.3)	
Do not know	4 (9.1)	1 (2.8)	
16. When you think about death and dying, how concerned are you that?			
16a. The family's money won't last:			0.289
Very concerned	2 (4.5)	1 (2.8)	
Concerned	10 (22.7)	10 (27.8)	
Neither concerned nor unconcerned	15 (34.1)	6 (16.7)	
Not concerned	6 (13.6)	7 (19.4)	
Not at all concerned	7 (15.9)	11 (30.6)	
Do not know	4 (9.1)	1 (2.8)	
16b. I will be a burden to, or overload, my family or friends.			0.394
Very concerned	7 (15.9)	6 (16.7)	
Concerned	18 (40.9)	9 (25.0)	
Neither concerned nor unconcerned	5 (11.4)	2 (5.6)	
Not concerned	8 (18.2)	11 (30.6)	
Not at all concerned	4 (9.1)	7 (19.4)	
Do not know	2 (4.5)	1 (2.8)	
17. Which of the following health problems, if any, do you think are worse than death? Select all that apply.[e]			
Living with great pain	21 (47.7)	12 (33.3)	0.193
Total physical dependency on others, for example, being in a wheelchair	15 (34.1)	8 (22.2)	0.243
Not being able to communicate my wishes and/or care to family members, for example, being in a coma Checked	27 (61.4)	21 (58.3)	0.783
None are worse than death	10 (22.7)	11 (30.6)	0.429
18. How important would each of the following be to you if you were dealing with your own dying?			
18a. Family/friends visiting you			0.512
Very important	40 (90.9)	29 (80.6)	
Somewhat important	3 (6.8)	5 (13.9)	
Neither important nor unimportant	1 (2.3)	1 (2.8)	
Do not know	0 (0.0)	1 (2.8)	

© 2020 Friebert S et al. *JAMA Network Open.*

18b. Being able to stay in your own home			0.231
**eTable 3. (continued) Frequencies of Adolescent Responses for Survey by Gender at Session 1 (N=80)**			
**Question**	**Female (N=44)**	**Male (N=36)**	**P-value[a]**
	**N (%)**	**N (%)**	
Very important	14 (31.8)	13 (36.1)	
Somewhat important	22 (50.0)	12 (33.3)	
Neither important nor unimportant	4 (9.1)	3 (8.3)	
Not very important	2 (4.5)	7 (19.4)	
Not at all important	1 (2.3)	1 (2.8)	
Do not know	1 (2.3)	0 (0.0)	
18c. Honest answers from your doctor			0.586
Very important	43 (97.7)	34 (94.4)	
Somewhat important	1 (2.3)	2 (5.6)	
18d. Comfort from church services or persons such as a minister, priest, imam, or rabbi			0.619
Very important	19 (43.2)	11 (30.6)	
Somewhat important	11 (25.0)	11 (30.6)	
Neither important nor unimportant	6 (13.6)	3 (8.3)	
Not very important	3 (6.8)	3 (8.3)	
Not at all important	5 (11.4)	7 (19.4)	
Do not know	0 (0.0)	1 (2.8)	
18e. Planning your own funeral			0.485
Very important	10 (22.7)	6 (16.7)	
Somewhat important	17 (38.6)	10 (27.8)	
Neither important nor unimportant	11 (25.0)	8 (22.2)	
Not very important	4 (9.1)	8 (22.2)	
Not at all important	1 (2.3)	2 (5.6)	
Do not know	1 (2.3)	2 (5.6)	0.429
18f. Being able to complete an advance directive that would let loved ones know your wishes, if you were unable to speak for yourself.			
Very important	26 (59.1)	17 (47.2)	
Somewhat important	12 (27.3)	16 (44.4)	
Neither important nor unimportant	3 (6.8)	1 (2.8)	
Do not know	3 (6.8)	2 (5.6)	
18g. Fulfilling personal goals/pleasures			0.354
Very important	27 (61.4)	28 (77.8)	
Somewhat important	15 (34.1)	7 (19.4)	
Neither important nor unimportant	1 (2.3)	1 (2.8)	
Not very important	1 (2.3)	0 (0.0)	
18h. Reviewing your life history with your family			0.492
Very important	18 (40.9)	16 (44.4)	

© 2020 Friebert S et al. *JAMA Network Open.*

Question	Female (N=44) N (%)	Male (N=36) N (%)	P-value[a]
Somewhat important	11 (25.0)	12 (33.3)	

eTable 3. (continued) Frequencies of Adolescent Responses for Survey by Gender at Session 1 (N=80)

Question	Female (N=44) N (%)	Male (N=36) N (%)	P-value[a]
Neither important nor unimportant	9 (20.5)	5 (13.9)	
Not very important	6 (13.6)	2 (5.6)	
Do not know	0 (0.0)	1 (2.8)	
18i. Having health care professionals visit you at your home			0.718
Very important	15 (34.1)	9 (25.0)	
Somewhat important	19 (43.2)	15 (41.7)	
Neither important nor unimportant	8 (18.2)	10 (27.8)	
Not very important	1 (2.3)	1 (2.8)	
Not at all important	0 (0.0)	1 (2.8)	
Do not know	1 (2.3)	0 (0.0)	
18j. Understanding your treatment choices			0.393
Very important	41 (93.2)	32 (88.9)	
Somewhat important	3 (6.8)	2 (5.6)	
Do not know	0 (0.0)	2 (5.6)	
19. How important are each of the following is to you when you think about dying?			
19a. Being physically comfortable			0.961
Very important	28 (63.6)	21 (58.3)	
Somewhat important	12 (27.3)	12 (33.3)	
Neither important nor unimportant	2 (4.5)	2 (5.6)	
Not very important	1 (2.3)	0 (0.0)	
Not at all important	1 (2.3)	1 (2.8)	
19b. Being free from pain			0.691
Very important	28 (63.6)	23 (63.9)	
Somewhat important	12 (27.3)	6 (16.7)	
Neither important nor unimportant	2 (4.5)	3 (8.3)	
Not very important	1 (2.3)	2 (5.6)	
Not at all important	1 (2.3)	1 (2.8)	
Do not know	0 (0.0)	1 (2.8)	
19c. Saying everything I want to say to people in my family			0.505
Very important	33 (76.7)	30 (83.3)	
Somewhat important	9 (20.9)	5 (13.9)	
Neither important nor unimportant	0 (0.0)	1 (2.8)	
Not at all important	1 (2.3)	0 (0.0)	
19d. Being at peace spiritually			0.521
Very important	35 (79.5)	24 (66.7)	
Somewhat important	7 (15.9)	8 (22.2)	

© 2020 Friebert S et al. *JAMA Network Open.*

Neither important nor unimportant	1 (2.3)	1 (2.8)	
Not very important	0 (0.0)	2 (5.6)	
Do not know	1 (2.3)	1 (2.8)	
19e. Not being a burden to loved ones			0.448
Very important	31 (70.5)	23 (63.9)	
Somewhat important	8 (18.2)	7 (19.4)	
Neither important nor unimportant	3 (6.8)	2 (5.6)	
Not very important	0 (0.0)	3 (8.3)	
Do not know	2 (4.5)	1 (2.8)	
19f. Knowing how to say good bye			0.020
Very important	37 (84.1)	22 (61.1)	
Somewhat important	4 (9.1)	8 (22.2)	
Neither important nor unimportant	3 (6.8)	1 (2.8)	
Not very important	0 (0.0)	2 (5.6)	
Do not know	0 (0.0)	3 (8.3)	
19g. Having a sense of my own worth or value			0.005
Very important	32 (72.7)	17 (47.2)	
Somewhat important	6 (13.6)	17 (47.2)	
Neither important nor unimportant	3 (6.8)	1 (2.8)	
Do not know	3 (6.8)	1 (2.8)	
19h. Being off machines that extend life, such as life support.			0.776
Very important	13 (29.5)	10 (27.8)	
Somewhat important	14 (31.8)	11 (30.6)	
Neither important nor unimportant	11 (25.0)	10 (27.8)	
Not very important	1 (2.3)	3 (8.3)	
Not at all important	2 (4.5)	0 (0.0)	
Do not know	3 (6.8)	2 (5.6)	
19i. Dying a natural death			0.095
Very important	15 (34.1)	10 (27.8)	
Somewhat important	7 (15.9)	16 (44.4)	
Neither important nor unimportant	14 (31.8)	6 (16.7)	
Not very important	4 (9.1)	2 (5.6)	
Not at all important	1 (2.3)	1 (2.8)	
Do not know	3 (6.8)	1 (2.8)	
20. If death were likely to happen in the next few weeks, and you could choose where to die, where would you MOST want to die?			0.426
At home without hospice	9 (20.5)	14 (38.9)	
At home in hospice	13 (29.5)	7 (19.4)	
In a hospital	6 (13.6)	4 (11.1)	
No preference	8 (18.2)	4 (11.1)	
Do not know	8 (18.2)	7 (19.4)	
21. Below are some statements related to pain near the end-of-life that have been expressed by people. How strongly do you agree or disagree with each statement?			

© 2020 Friebert S et al. *JAMA Network Open.*

Question	Female (N=44) N (%)	Male (N=36) N (%)	P-value[a]
21a. I am afraid the doctor may not believe I am in pain and treat my pain			0.878
**eTable 3. (continued) Frequencies of Adolescent Responses for Survey by Gender at Session 1 (N=80)**			
Strongly agree	1 (2.3)	1 (2.8)	
Agree	8 (18.2)	5 (13.9)	
Neither agree or disagree	5 (11.4)	5 (13.9)	
Disagree	18 (40.9)	12 (33.3)	
Strongly disagree	10 (22.7)	9 (25.0)	
Do not know	2 (4.5)	4 (11.1)	
21b. I would only take pain medicines when the pain is severe			0.533
Strongly agree	14 (31.8)	11 (30.6)	
Agree	16 (36.4)	12 (33.3)	
Neither agree or disagree	7 (15.9)	3 (8.3)	
Disagree	6 (13.6)	9 (25.0)	
Strongly disagree	1 (2.3)	0 (0.0)	
Do not know	0 (0.0)	1 (2.8)	
21c. I am afraid I will become addicted to the pain medicines over time			0.708
Strongly agree	2 (4.5)	1 (2.8)	
Agree	10 (22.7)	7 (19.4)	
Neither agree or disagree	2 (4.5)	3 (8.3)	
Disagree	12 (27.3)	12 (33.3)	
Strongly disagree	17 (38.6)	10 (27.8)	
Do not know	1 (2.3)	3 (8.3)	
21d. I would take the lowest amount of medicine possible to save larger doses for later when the pain is worse			0.297
Strongly agree	10 (22.7)	5 (13.9)	
Agree	19 (43.2)	12 (33.3)	
Neither agree or disagree	7 (15.9)	5 (13.9)	
Disagree	5 (11.4)	6 (16.7)	
Strongly disagree	3 (6.8)	5 (13.9)	
Do not know	0 (0.0)	3 (8.3)	
21e. I am afraid I would be given too much pain medicine			0.305
Strongly agree	2 (4.5)	4 (11.1)	
Agree	7 (15.9)	7 (19.4)	
Neither agree or disagree	7 (15.9)	3 (8.3)	
Disagree	18 (40.9)	11 (30.6)	
Strongly disagree	10 (22.7)	8 (22.2)	
Do not know	0 (0.0)	3 (8.3)	
22. Have you heard of hospice services?			0.695

© 2020 Friebert S et al. *JAMA Network Open.*

Yes	30 (68.2)	26 (72.2)	
No	14 (31.8)	10 (27.8)	

eTable 3. (continued) Frequencies of Adolescent Responses for Survey by Gender at Session 1 (N=80)

Question	Female (N=44) N (%)	Male (N=36) N (%)	P-value[a]
23. How did you learn about hospice services? (N=56)			0.472
I know someone who used hospice services	18 (60.0)	11 (42.3)	
I heard from a health care professional	1 (3.3)	3 (11.5)	
I read literature/newspaper/TV/radio/other media	3 (10.0)	4 (15.4)	
I heard from others	8 (26.7)	8 (30.8)	
24. If you were dying, would you want hospice support? (N=56)			0.147
Yes	13 (43.3)	11 (42.3)	
No	1 (3.3)	5 (19.2)	
Don't know/not sure	16 (53.3)	10 (38.5)	
25. Do you consider yourself religious/spiritual?			0.203
Very religious/spiritual	13 (29.5)	8 (22.2)	
Somewhat religious/spiritual	20 (45.5)	19 (52.8)	
Not very religious/spiritual	7 (15.9)	2 (5.6)	
Not religious/spiritual	3 (6.8)	7 (19.4)	
Declined	1 (2.3)	0 (0.0)	
26. How often do you attend religious or spiritual services? (N=69)			0.874
Always	4 (10.0)	2 (6.9)	
Very often	9 (22.5)	7 (24.1)	
Sometimes	18 (45.0)	14 (48.3)	
Rarely	8 (20.0)	4 (13.8)	
Never	1 (2.5)	2 (6.9)	
27. How often does religion or spirituality help you face your fears or do what you were afraid to do? (N=69)			0.358
Everyday	9 (22.5)	9 (31.0)	
A few times a week	8 (20.0)	9 (31.0)	
A few times a month	12 (30.0)	6 (20.7)	
Rarely	7 (17.5)	5 (17.2)	
Never	4 (10.0)	0 (0.0)	
30. HOW MANY NIGHTS did you stay in a hospital?			0.706
0	38 (86.4)	33 (91.7)	
1-2	1 (2.3)	0 (0.0)	
3-5	3 (6.8)	1 (2.8)	
6-10	1 (2.3)	2 (5.6)	
>20	1 (2.3)	0 (0.0)	
31. How healthy are you feeling right now?			0.696
Excellent health	10 (22.7)	12 (33.3)	

© 2020 Friebert S et al. *JAMA Network Open.*

Very good health	17 (38.6)	14 (38.9)	

eTable 3. (continued) Frequencies of Adolescent Responses for Survey by Gender at Session 1 (N=80)

Question	Female (N=44)	Male (N=36)	P-value[a]
	N (%)	N (%)	
Good health	10 (22.7)	5 (13.9)	
Fair health	6 (13.6)	3 (8.3)	
Poor health	1 (2.3)	1 (2.8)	
Declined	0 (0.0)	1 (2.8)	
32a. Is your mother alive?			1.000
Yes	43 (97.7)	36 (100.0)	
No	1 (2.3)	0 (0.0)	
32b. Is your father alive?			0.685
Yes	39 (88.6)	34 (94.4)	
No	3 (6.8)	2 (5.6)	
Do not know	2 (4.5)	0 (0.0)	

[a:] Pearson chi-square test or Fisher' exact test.
[b]: Bonferroni corrected significant level is 0.05/8=0.006.
[c]: Bonferroni corrected significant level is 0.05/4=0.013
[d]: Bonferroni corrected significant level is 0.05/10=0.005.
[e]: Bonferroni corrected significant level is 0.05/4=0.013

© 2020 Friebert S et al. *JAMA Network Open.*

eTable 4. Frequencies of Adolescent Responses for Survey by Race at Session 1 (N=79[a])			
Question	White (N=60)	Non-White (N=19)	P-value[b]
	N (%)	N (%)	
1. Have you ever written down any thoughts about your future health plans?			0.280
Yes, definitely	1 (1.7)	2 (10.5)	
Very probably	1 (1.7)	0 (0.0)	
Probably	5 (8.3)	0 (0.0)	
Probably not	7 (11.7)	4 (21.1)	
Definitely no	43 (71.7)	12 (63.2)	
Do not know	3 (5.0)	1 (5.3)	
2a. Have you ever heard about and completed a Health Care Power of Attorney (HCPA), in which you name someone to make decisions about your health care in case you could not?			0.588
Have heard about and completed	1 (1.7)	0 (0.0)	
Have heard about but not completed	27 (45.0)	9 (47.4)	
Have not heard about	28 (46.7)	7 (36.8)	
Do not know	4 (6.7)	3 (15.8)	
2b. Have you ever heard about and completed an Advance Directive or living will, such as the Five Wishes?			0.650
Have heard about and completed	2 (3.3)	0 (0.0)	
Have heard about but not completed	24 (40.0)	5 (26.3)	
Have not heard about	31 (51.7)	13 (68.4)	
Do not know	3 (5.0)	1 (5.3)	
3a. Whether you have completed any advance directives/pre-plans or not, have you talked about your wishes for care at the end of life with anyone? Select all that apply.[c]			
Spouse/partner	0 (0.0)	0 (0.0)	-
Parents	18 (30.0)	6 (31.6)	0.896
Siblings (brother/sister)	8 (13.3)	1 (5.3)	0.679
Friends	8 (13.3)	0 (0.0)	0.188
Boyfriend/girlfriend	3 (5.0)	1 (5.3)	1.000
Lawyer	0 (0.0)	0 (0.0)	-
Primary physician	1 (1.7)	0 (0.0)	1.000
Clergy (such as minister, rabbi, iman etc.)	1 (1.7)	0 (0.0)	1.000
Other	0 (0.0)	1 (5.3)	0.241
Have not talked with anyone	38 (63.3)	12 (63.2)	0.989
3b. Do you have any children?			
No	60 (100.0)	19 (100.0)	-
4. If you were very ill and knew that you would not get better, who would you want to be involved in decisions about your end-of-life care? Select all that apply.[d]			
Myself	39 (65.0)	13 (68.4)	0.784

© 2020 Friebert S et al. *JAMA Network Open.*

**eTable 4. (continued) Frequencies of Adolescent Responses for Survey by Race at Session 1 (N=79[a])**			
**Question**	**White (N=60)**	**Non-White (N=19)**	**P-value[b]**
	**N (%)**	**N (%)**	
My family	58 (96.7)	18 (94.7)	0.567
My doctor	34 (56.7)	11 (57.9)	0.925
Someone else	6 (10.0)	2 (10.5)	1.000
5. When do you think it is the best time to bring up end-of-life decisions?			0.059
Before getting sick, while healthy	27 (45.0)	4 (21.1)	
When first diagnosed with a life-threatening illness	7 (11.7)	4 (21.1)	
When first sick from a life-threatening illness	4 (6.7)	2 (10.5)	
When first hospitalized with a life-threatening illness	0 (0.0)	1 (5.3)	
If dying	4 (6.7)	0 (0.0)	
All of the above	1 (1.7)	2 (10.5)	
Never	15 (25.0)	5 (26.3)	
Other	0 (0.0)	1 (5.3)	
Do not know	2 (3.3)	0 (0.0)	
6. Who are the best people/best person on the treatment team to bring it up with you? Select all that apply.[e]			
Physician	37 (61.7)	12 (63.2)	0.907
Nurse practitioner	21 (35.0)	5 (26.3)	0.483
Nurse	19 (31.7)	5 (26.3)	0.659
Social worker	18 (30.0)	4 (21.1)	0.564
Psychologist	11 (18.3)	2 (10.5)	0.723
Case manager	3 (5.0)	1 (5.3)	1.000
Chaplain	4 (6.7)	1 (5.3)	1.000
Patient advocate	6 (10.0)	1 (5.3)	1.000
Other	3 (5.0)	1 (5.3)	1.000
Do not know	9 (15.0)	2 (10.5)	1.000
7. Do you believe that once you make an important medical decision, for example, to be put on a respirator, a machine that breathes for you, that you would be able to change your mind?			0.105
Yes, definitely	11 (18.3)	8 (42.1)	
Very probably	11 (18.3)	0 (0.0)	
Probably	21 (35.0)	5 (26.3)	
Probably not	11 (18.3)	4 (21.1)	
Do not know	6 (10.0)	2 (10.5)	
8. Do you think your doctor or the hospital will respect your wishes, that is, do what you want about medical care?			1.000
Yes, definitely	30 (50.0)	10 (52.6)	
Very probably	15 (25.0)	5 (26.3)	
Probably	9 (15.0)	3 (15.8)	

© 2020 Friebert S et al. *JAMA Network Open.*

**eTable 4. (continued) Frequencies of Adolescent Responses for Survey by Race at Session 1 (N=79[a])**			
**Question**	**White (N=60)**	**Non-White (N=19)**	**P-value[b]**
	**N (%)**	**N (%)**	
Probably not	3 (5.0)	1 (5.3)	
Do not know	3 (5.0)	0 (0.0)	
9. Do you think your parent/guardian/surrogate understands your wishes?			0.891
Yes, definitely	29 (48.3)	12 (66.7)	
Very probably	11 (18.3)	2 (11.1)	
Probably	12 (20.0)	3 (16.7)	
Probably not	4 (6.7)	1 (5.6)	
Definitely no	1 (1.7)	0 (0.0)	
Do not know	3 (5.0)	0 (0.0)	
10. Do you think your parent/guardian/surrogate will respect your wishes, that is, do what you want about your medical care?			1.000
Yes, definitely	31 (51.7)	10 (52.6)	
Very probably	13 (21.7)	4 (21.1)	
Probably	11 (18.3)	3 (15.8)	
Probably not	3 (5.0)	1 (5.3)	
Do not know	2 (3.3)	1 (5.3)	
11. How often has death and dying been talked about in your family?			0.363
Very often	2 (3.3)	0 (0.0)	
Often	6 (10.0)	6 (31.6)	
Occasionally	20 (33.3)	5 (26.3)	
Rarely	25 (41.7)	7 (36.8)	
Never	4 (6.7)	1 (5.3)	
Do not know	3 (5.0)	0 (0.0)	
12. How comfortable are you talking about death?			0.797
Very comfortable	13 (21.7)	3 (15.8)	
Somewhat comfortable	25 (41.7)	8 (42.1)	
Neither comfortable or uncomfortable	12 (20.0)	4 (21.1)	
Not very comfortable	6 (10.0)	1 (5.3)	
Not at all comfortable	3 (5.0)	2 (10.5)	
Do not know	1 (1.7)	1 (5.3)	
13. How likely are you to?			
13a. Attend funerals or memorial services when a loved one, friend or classmate dies			0.056
Very likely	47 (78.3)	13 (68.4)	
Somewhat likely	10 (16.7)	2 (10.5)	
Neither likely or unlikely	0 (0.0)	2 (10.5)	
Not very likely	0 (0.0)	1 (5.3)	

© 2020 Friebert S et al. *JAMA Network Open.*

eTable 4. (continued) Frequencies of Adolescent Responses for Survey by Race at Session 1 (N=79[a])			
Question	White (N=60)	Non-White (N=19)	P-value[b]
	N (%)	N (%)	
Not at all likely	1 (1.7)	1 (5.3)	
Do not know	2 (3.3)	0 (0.0)	
13b. Avoid medical checkups because you are afraid the doctor will find "something serious"			0.710
Very likely	2 (3.3)	0 (0.0)	
Somewhat likely	9 (15.0)	1 (5.3)	
Neither likely or unlikely	3 (5.0)	0 (0.0)	
Not very likely	8 (13.3)	4 (21.1)	
Not at all likely	37 (61.7)	14 (73.7)	
Do not know	1 (1.7)	0 (0.0)	
13c. Speak freely to loved ones about death and dying			0.677
Very likely	11 (18.3)	6 (31.6)	
Somewhat likely	23 (38.3)	5 (26.3)	
Neither likely or unlikely	13 (21.7)	4 (21.1)	
Not very likely	7 (11.7)	1 (5.3)	
Not at all likely	5 (8.3)	3 (15.8)	
Do not know	1 (1.7)	0 (0.0)	
13d. Visit or telephone a friend or relative who has recently lost a loved one in order to see how they are doing			1.000
Very likely	44 (73.3)	14 (73.7)	
Somewhat likely	12 (20.0)	4 (21.1)	
Neither likely or unlikely	2 (3.3)	1 (5.3)	
Not very likely	2 (3.3)	0 (0.0)	
13e. Preplan your own funeral, for example, choose someone to speak or choose the music you would want			0.258
Very likely	22 (36.7)	4 (21.1)	
Somewhat likely	10 (16.7)	4 (21.1)	
Neither likely or unlikely	9 (15.0)	4 (21.1)	
Not very likely	5 (8.3)	5 (26.3)	
Not at all likely	10 (16.7)	1 (5.3)	
Do not know	4 (6.7)	1 (5.3)	
14. How afraid, if at all, are you of?			
14a. Dying from a long term illness			0.166
Very afraid	5 (8.3)	4 (21.1)	
Somewhat afraid	22 (36.7)	3 (15.8)	
Neither afraid nor not afraid	7 (11.7)	2 (10.5)	
Not very afraid	10 (16.7)	7 (36.8)	
Not at all afraid	13 (21.7)	3 (15.8)	
Do not know	3 (5.0)	0 (0.0)	

© 2020 Friebert S et al. *JAMA Network Open.*

**eTable 4. (continued) Frequencies of Adolescent Responses for Survey by Race at Session 1 (N=79[a])**			
**Question**	**White (N=60)**	**Non-White (N=19)**	**P-value[b]**
	**N (%)**	**N (%)**	
14b. Dying suddenly, such as an accident, or being killed:			0.832
Very afraid	10 (16.7)	4 (21.1)	
Somewhat afraid	21 (35.0)	4 (21.1)	
Neither afraid nor not afraid	9 (15.0)	4 (21.1)	
Not very afraid	10 (16.7)	4 (21.1)	
Not at all afraid	9 (15.0)	3 (15.8)	
Do not know	1 (1.7)	0 (0.0)	
14c. Dying alone			0.888
Very afraid	18 (30.0)	6 (31.6)	
Somewhat afraid	10 (16.7)	4 (21.1)	
Neither afraid nor not afraid	10 (16.7)	3 (15.8)	
Not very afraid	9 (15.0)	1 (5.3)	
Not at all afraid	12 (20.0)	5 (26.3)	
Do not know	1 (1.7)	0 (0.0)	
14d. Dying in an institution such as a nursing home or hospital			0.036
Very afraid	7 (11.7)	6 (31.6)	
Somewhat afraid	10 (16.7)	2 (10.5)	
Neither afraid nor not afraid	14 (23.3)	1 (5.3)	
Not very afraid	14 (23.3)	1 (5.3)	
Not at all afraid	13 (21.7)	8 (42.1)	
Do not know	2 (3.3)	1 (5.3)	
14e. Dying painfully			0.069
Very afraid	17 (28.3)	10 (52.6)	
Somewhat afraid	24 (40.0)	2 (10.5)	
Neither afraid nor not afraid	6 (10.0)	1 (5.3)	
Not very afraid	7 (11.7)	2 (10.5)	
Not at all afraid	5 (8.3)	4 (21.1)	
Do not know	1 (1.7)	0 (0.0)	
15. How strongly do you agree or disagree that?			
15a. Dying is an important part of life			0.776
Strongly agree	24 (40.0)	9 (47.4)	
Agree	24 (40.0)	5 (26.3)	
Neither agree or disagree	6 (10.0)	3 (15.8)	
Disagree	4 (6.7)	2 (10.5)	
Strongly disagree	1 (1.7)	0 (0.0)	
Do not know	1 (1.7)	0 (0.0)	
15b. If someone could tell me when I would die, I would want to know.			0.222

© 2020 Friebert S et al. *JAMA Network Open.*

**eTable 4. (continued) Frequencies of Adolescent Responses for Survey by Race at Session 1 (N=79[a])**			
**Question**	**White (N=60)**	**Non-White (N=19)**	**P-value[b]**
	**N (%)**	**N (%)**	
Strongly agree	9 (15.0)	7 (36.8)	
Agree	12 (20.0)	1 (5.3)	
Neither agree or disagree	8 (13.3)	2 (10.5)	
Disagree	7 (11.7)	4 (21.1)	
Strongly disagree	20 (33.3)	4 (21.1)	
Do not know	4 (6.7)	1 (5.3)	
16. When you think about death and dying, how concerned are you that?			
16a. The family's money won't last:			0.614
Very concerned	2 (3.3)	1 (5.3)	
Concerned	17 (28.3)	3 (15.8)	
Neither concerned nor unconcerned	14 (23.3)	7 (36.8)	
Not concerned	8 (13.3)	4 (21.1)	
Not at all concerned	15 (25.0)	3 (15.8)	
Do not know	4 (6.7)	1 (5.3)	
16b. I will be a burden to, or overload, my family or friends.			0.760
Very concerned	8 (13.3)	5 (26.3)	
Concerned	21 (35.0)	5 (26.3)	
Neither concerned nor unconcerned	6 (10.0)	1 (5.3)	
Not concerned	14 (23.3)	5 (26.3)	
Not at all concerned	8 (13.3)	3 (15.8)	
Do not know	3 (5.0)	0 (0.0)	
17. Which of the following health problems, if any, do you think are worse than death? Select all that apply.[f]			
Living with great pain	22 (36.7)	11 (57.9)	0.102
Total physical dependency on others, for example, being in a wheelchair	15 (25.0)	7 (36.8)	0.316
Not being able to communicate my wishes and/or care to family members, for example, being in a coma Checked	36 (60.0)	12 (63.2)	0.806
None are worse than death	17 (28.3)	4 (21.1)	0.767
18. How important would each of the following be to you if you were dealing with your own dying?			
18a. Family/friends visiting you			0.097
Very important	53 (88.3)	15 (78.9)	
Somewhat important	6 (10.0)	2 (10.5)	
Neither important nor unimportant	0 (0.0)	2 (10.5)	
Do not know	1 (1.7)	0 (0.0)	
18b. Being able to stay in your own home			0.942
Very important	19 (31.7)	8 (42.1)	

© 2020 Friebert S et al. *JAMA Network Open.*

eTable 4. (continued) Frequencies of Adolescent Responses for Survey by Race at Session 1 (N=79[a])			
Question	White (N=60)	Non-White (N=19)	P-value[b]
	N (%)	N (%)	
Somewhat important	27 (45.0)	7 (36.8)	
Neither important nor unimportant	5 (8.3)	2 (10.5)	
Not very important	6 (10.0)	2 (10.5)	
Not at all important	2 (3.3)	0 (0.0)	
Do not know	1 (1.7)	0 (0.0)	
18c. Honest answers from your doctor			0.567
Very important	58 (96.7)	18 (94.7)	
Somewhat important	2 (3.3)	1 (5.3)	
18d. Comfort from church services or persons such as a minister, priest, imam, or rabbi			0.957
Very important	21 (35.0)	9 (47.4)	
Somewhat important	16 (26.7)	5 (26.3)	
Neither important nor unimportant	7 (11.7)	2 (10.5)	
Not very important	5 (8.3)	1 (5.3)	
Not at all important	10 (16.7)	2 (10.5)	
Do not know	1 (1.7)	0 (0.0)	
18e. Planning your own funeral			0.277
Very important	11 (18.3)	5 (26.3)	
Somewhat important	22 (36.7)	4 (21.1)	
Neither important nor unimportant	11 (18.3)	8 (42.1)	
Not very important	10 (16.7)	2 (10.5)	
Not at all important	3 (5.0)	0 (0.0)	
Do not know	3 (5.0)	0 (0.0)	
18f. Being able to complete an advance directive that would let loved ones know your wishes, if you were unable to speak for yourself.			0.259
Very important	33 (55.0)	9 (47.4)	
Somewhat important	22 (36.7)	6 (31.6)	
Neither important nor unimportant	3 (5.0)	1 (5.3)	
Do not know	2 (3.3)	3 (15.8)	
18g. Fulfilling personal goals/pleasures			1.000
Very important	40 (66.7)	14 (73.7)	
Somewhat important	17 (28.3)	5 (26.3)	
Neither important nor unimportant	2 (3.3)	0 (0.0)	
Not very important	1 (1.7)	0 (0.0)	
18h. Reviewing your life history with your family			0.170
Very important	22 (36.7)	11 (57.9)	
Somewhat important	20 (33.3)	3 (15.8)	
Neither important nor unimportant	11 (18.3)	3 (15.8)	

© 2020 Friebert S et al. *JAMA Network Open.*

eTable 4. (continued) Frequencies of Adolescent Responses for Survey by Race at Session 1 (N=79[a])			
Question	White (N=60)	Non-White (N=19)	P-value[b]
	N (%)	N (%)	
Not very important	7 (11.7)	1 (5.3)	
Do not know	0 (0.0)	1 (5.3)	
18i. Having health care professionals visit you at your home			1.000
Very important	18 (30.0)	6 (31.6)	
Somewhat important	25 (41.7)	8 (42.1)	
Neither important nor unimportant	13 (21.7)	5 (26.3)	
Not very important	2 (3.3)	0 (0.0)	
Not at all important	1 (1.7)	0 (0.0)	
Do not know	1 (1.7)	0 (0.0)	
18j. Understanding your treatment choices			0.092
Very important	57 (95.0)	15 (78.9)	
Somewhat important	2 (3.3)	3 (15.8)	
Do not know	1 (1.7)	1 (5.3)	
19. How important are each of the following is to you when you think about dying?			
19a. Being physically comfortable			0.559
Very important	36 (60.0)	13 (68.4)	
Somewhat important	20 (33.3)	4 (21.1)	
Neither important nor unimportant	2 (3.3)	1 (5.3)	
Not very important	1 (1.7)	0 (0.0)	
Not at all important	1 (1.7)	1 (5.3)	
19b. Being free from pain			0.419
Very important	39 (65.0)	12 (63.2)	
Somewhat important	14 (23.3)	4 (21.1)	
Neither important nor unimportant	3 (5.0)	1 (5.3)	
Not very important	3 (5.0)	0 (0.0)	
Not at all important	1 (1.7)	1 (5.3)	
Do not know	0 (0.0)	1 (5.3)	
19c. Saying everything I want to say to people in my family			0.058
Very important	44 (73.3)	18 (100.0)	
Somewhat important	14 (23.3)	0 (0.0)	
Neither important nor unimportant	1 (1.7)	0 (0.0)	
Not at all important	1 (1.7)	0 (0.0)	
19d. Being at peace spiritually			0.784
Very important	43 (71.7)	15 (78.9)	
Somewhat important	12 (20.0)	3 (15.8)	
Neither important nor unimportant	1 (1.7)	1 (5.3)	
Not very important	2 (3.3)	0 (0.0)	

© 2020 Friebert S et al. *JAMA Network Open.*

eTable 4. (continued) Frequencies of Adolescent Responses for Survey by Race at Session 1 (N=79[a])			
Question	White (N=60)	Non-White (N=19)	P-value[b]
	N (%)	N (%)	
Do not know	2 (3.3)	0 (0.0)	
19e. Not being a burden to loved ones			0.467
Very important	38 (63.3)	16 (84.2)	
Somewhat important	12 (20.0)	2 (10.5)	
Neither important nor unimportant	5 (8.3)	0 (0.0)	
Not very important	2 (3.3)	1 (5.3)	
Do not know	3 (5.0)	0 (0.0)	
19f. Knowing how to say good bye			0.438
Very important	45 (75.0)	13 (68.4)	
Somewhat important	9 (15.0)	3 (15.8)	
Neither important nor unimportant	3 (5.0)	1 (5.3)	
Not very important	2 (3.3)	0 (0.0)	
Do not know	1 (1.7)	2 (10.5)	
19g. Having a sense of my own worth or value			0.417
Very important	35 (58.3)	14 (73.7)	
Somewhat important	20 (33.3)	3 (15.8)	
Neither important nor unimportant	2 (3.3)	1 (5.3)	
Do not know	3 (5.0)	1 (5.3)	
19h. Being off machines that extend life, such as life support.			0.649
Very important	17 (28.3)	6 (31.6)	
Somewhat important	20 (33.3)	5 (26.3)	
Neither important nor unimportant	16 (26.7)	4 (21.1)	
Not very important	2 (3.3)	2 (10.5)	
Not at all important	1 (1.7)	1 (5.3)	
Do not know	4 (6.7)	1 (5.3)	
19i. Dying a natural death			0.295
Very important	18 (30.0)	7 (36.8)	
Somewhat important	15 (25.0)	8 (42.1)	
Neither important nor unimportant	16 (26.7)	3 (15.8)	
Not very important	6 (10.0)	0 (0.0)	
Not at all important	1 (1.7)	1 (5.3)	
Do not know	4 (6.7)	0 (0.0)	
20. If death were likely to happen in the next few weeks, and you could choose where to die, where would you MOST want to die?			0.484
At home without hospice	16 (26.7)	7 (36.8)	
At home in hospice	16 (26.7)	3 (15.8)	
In a hospital	6 (10.0)	4 (21.1)	
No preference	9 (15.0)	3 (15.8)	

© 2020 Friebert S et al. *JAMA Network Open.*

eTable 4. (continued) Frequencies of Adolescent Responses for Survey by Race at Session 1 (N=79[a])			
Question	White (N=60)	Non-White (N=19)	P-value[b]
	N (%)	N (%)	
Do not know	13 (21.7)	2 (10.5)	
21. Below are some statements related to pain near the end-of-life that have been expressed by people. How strongly do you agree or disagree with each statement?			
21a. I am afraid the doctor may not believe I am in pain and treat my pain			0.451
Strongly agree	2 (3.3)	0 (0.0)	
Agree	8 (13.3)	5 (26.3)	
Neither agree or disagree	9 (15.0)	1 (5.3)	
Disagree	24 (40.0)	5 (26.3)	
Strongly disagree	13 (21.7)	6 (31.6)	
Do not know	4 (6.7)	2 (10.5)	
21b. I would only take pain medicines when the pain is severe			0.788
Strongly agree	17 (28.3)	8 (42.1)	
Agree	23 (38.3)	5 (26.3)	
Neither agree or disagree	7 (11.7)	3 (15.8)	
Disagree	11 (18.3)	3 (15.8)	
Strongly disagree	1 (1.7)	0 (0.0)	
Do not know	1 (1.7)	0 (0.0)	
21c. I am afraid I will become addicted to the pain medicines over time			0.437
Strongly agree	2 (3.3)	1 (5.3)	
Agree	11 (18.3)	6 (31.6)	
Neither agree or disagree	5 (8.3)	0 (0.0)	
Disagree	19 (31.7)	4 (21.1)	
Strongly disagree	19 (31.7)	8 (42.1)	
Do not know	4 (6.7)	0 (0.0)	
21d. I would take the lowest amount of medicine possible to save larger doses for later when the pain is worse			0.647
Strongly agree	11 (18.3)	4 (21.1)	
Agree	23 (38.3)	8 (42.1)	
Neither agree or disagree	11 (18.3)	1 (5.3)	
Disagree	7 (11.7)	3 (15.8)	
Strongly disagree	5 (8.3)	3 (15.8)	
Do not know	3 (5.0)	0 (0.0)	
21e. I am afraid I would be given too much pain medicine			0.711
Strongly agree	4 (6.7)	2 (10.5)	
Agree	9 (15.0)	5 (26.3)	
Neither agree or disagree	7 (11.7)	3 (15.8)	

© 2020 Friebert S et al. *JAMA Network Open.*

eTable 4. (continued) Frequencies of Adolescent Responses for Survey by Race at Session 1 (N=79[a])			
Question	White (N=60)	Non-White (N=19)	P-value[b]
	N (%)	N (%)	
Disagree	23 (38.3)	5 (26.3)	
Strongly disagree	14 (23.3)	4 (21.1)	
Do not know	3 (5.0)	0 (0.0)	
22. Have you heard of hospice services?			0.016
Yes	46 (76.7)	9 (47.4)	
No	14 (23.3)	10 (52.6)	
23. How did you learn about hospice services? (N=56)			0.139
I know someone who used hospice services	25 (54.3)	3 (33.3)	
I heard from a health care professional	2 (4.3)	2 (22.2)	
I read literature/newspaper/TV/radio/other media	5 (10.9)	2 (22.2)	
I heard from others	14 (30.4)	2 (22.2)	
24. If you were dying, would you want hospice support? (N=56)			0.113
Yes	20 (43.5)	3 (33.3)	
No	3 (6.5)	3 (33.3)	
Don't know/not sure	23 (50.0)	3 (33.3)	
25. Do you consider yourself religious/spiritual?			0.198
Very religious/spiritual	14 (23.3)	7 (36.8)	
Somewhat religious/spiritual	31 (51.7)	7 (36.8)	
Not very religious/spiritual	6 (10.0)	3 (15.8)	
Not religious/spiritual	9 (15.0)	1 (5.3)	
Declined	0 (0.0)	1 (5.3)	
26. How often do you attend religious or spiritual services? (N=69)			0.802
Always	3 (5.9)	2 (11.8)	
Very often	13 (25.5)	3 (17.6)	
Sometimes	23 (45.1)	9 (52.9)	
Rarely	9 (17.6)	3 (17.6)	
Never	3 (5.9)	0 (0.0)	
27. How often does religion or spirituality help you face your fears or do what you were afraid to do? (N=69)			0.118
Everyday	10 (19.6)	8 (47.1)	
A few times a week	15 (29.4)	2 (11.8)	
A few times a month	14 (27.5)	3 (17.6)	
Rarely	10 (19.6)	2 (11.8)	
Never	2 (3.9)	2 (11.8)	
30. HOW MANY NIGHTS did you stay in a hospital?			0.097
0	56 (93.3)	15 (78.9)	
1-2	0 (0.0)	1 (5.3)	

© 2020 Friebert S et al. *JAMA Network Open.*

eTable 4. (continued) Frequencies of Adolescent Responses for Survey by Race at Session 1 (N=79[a])			
Question	White (N=60)	Non-White (N=19)	P-value[b]
	N (%)	N (%)	
3-5	2 (3.3)	1 (5.3)	
6-10	2 (3.3)	1 (5.3)	
>20	0 (0.0)	1 (5.3)	
31. How healthy are you feeling right now?			0.643
Excellent health	15 (25.0)	7 (36.8)	
Very good health	26 (43.3)	5 (26.3)	
Good health	10 (16.7)	5 (26.3)	
Fair health	6 (10.0)	2 (10.5)	
Poor health	2 (3.3)	0 (0.0)	
Declined	1 (1.7)	0 (0.0)	
32a. Is your mother alive?			1.000
Yes	59 (98.3)	19 (100.0)	
No	1 (1.7)	0 (0.0)	
32b. Is your father alive?			0.232
Yes	56 (93.3)	16 (84.2)	
No	3 (5.0)	2 (10.5)	
Do not know	1 (1.7)	1 (5.3)	

[a]: One unknown race was excluded.
[b]: Pearson chi-square test or Fisher' exact test.
[c]: Bonferroni corrected significant level is 0.05/8=0.006.
[d]: Bonferroni corrected significant level is 0.05/4=0.013.
[e]: Bonferroni corrected significant level is 0.05/10=0.005.
[f]: Bonferroni corrected significant level is 0.05/4=0.013.

© 2020 Friebert S et al. *JAMA Network Open.*

eTable 5. Frequencies of Adolescent Responses for Survey by Poverty at Session 1 (N=78[a])

Question	Poverty (N=21)	Non-Poverty (N=57)	P-value[b]
	N (%)	N (%)	
1. Have you ever written down any thoughts about your future health plans?			0.294
Yes, definitely	2 (9.5)	1 (1.8)	
Very probably	1 (4.8)	0 (0.0)	
Probably	1 (4.8)	4 (7.0)	
Probably not	3 (14.3)	8 (14.0)	
Definitely no	13 (61.9)	42 (73.7)	
Do not know	1 (4.8)	2 (3.5)	
2a. Have you ever heard about and completed a Health Care Power of Attorney (HCPA), in which you name someone to make decisions about your health care in case you could not?			0.347
Have heard about and completed	0 (0.0)	2 (3.5)	
Have heard about but not completed	10 (47.6)	25 (43.9)	
Have not heard about	11 (52.4)	23 (40.4)	
Do not know	0 (0.0)	7 (12.3)	
2b. Have you ever heard about and completed an Advance Directive or living will, such as the Five Wishes?			0.800
Have heard about and completed	0 (0.0)	2 (3.5)	
Have heard about but not completed	10 (47.6)	20 (35.1)	
Have not heard about	10 (47.6)	32 (56.1)	
Do not know	1 (4.8)	3 (5.3)	
3a. Whether you have completed any advance directives/pre-plans or not, have you talked about your wishes for care at the end of life with anyone? Select all that apply.[c]			
Spouse/partner	0 (0.0)	0 (0.0)	-
Parents	5 (23.8)	20 (35.1)	0.344
Siblings (brother/sister)	3 (14.3)	6 (10.5)	0.696
Friends	3 (14.3)	6 (10.5)	0.696
Boyfriend/girlfriend	2 (9.5)	2 (3.5)	0.292
Lawyer	0 (0.0)	0 (0.0)	-
Primary physician	0 (0.0)	1 (1.8)	1.000
Clergy (such as minister, rabbi, iman etc.)	0 (0.0)	1 (1.8)	1.000
Other	0 (0.0)	1 (1.8)	1.000
Have not talked with anyone	14 (66.7)	34 (59.6)	0.572
3b. Do you have any children?			
No	21 (100.0)	57 (100.0)	-
4. If you were very ill and knew that you would not get better, who would you want to be involved in decisions about your end-of-life care? Select all that apply.[d]			

© 2020 Friebert S et al. *JAMA Network Open.*

eTable 5. (continued) Frequencies of Adolescent Responses for Survey by Poverty at Session 1 (N=78[a])			
**Question**	**Poverty (N=21)**	**Non-Poverty (N=57)**	**P-value[b]**
	**N (%)**	**N (%)**	
Myself	13 (61.9)	38 (66.7)	0.695
My family	20 (95.2)	55 (96.5)	1.000
My doctor	11 (52.4)	33 (57.9)	0.663
Someone else	3 (14.3)	4 (7.0)	0.379
5. When do you think it is the best time to bring up end-of-life decisions?			0.626
Before getting sick, while healthy	8 (38.1)	23 (40.4)	
When first diagnosed with a life-threatening illness	4 (19.0)	7 (12.3)	
When first sick from a life-threatening illness	2 (9.5)	4 (7.0)	
When first hospitalized with a life-threatening illness	0 (0.0)	1 (1.8)	
If dying	0 (0.0)	4 (7.0)	
All of the above	1 (4.8)	2 (3.5)	
Never	4 (19.0)	15 (26.3)	
Other	1 (4.8)	0 (0.0)	
Do not know	1 (4.8)	1 (1.8)	
6. Who are the best people/best person on the treatment team to bring it up with you? Select all that apply.[e]			
Physician	12 (57.1)	36 (63.2)	0.628
Nurse practitioner	5 (23.8)	20 (35.1)	0.344
Nurse	7 (33.3)	16 (28.1)	0.651
Social worker	5 (23.8)	18 (31.6)	0.505
Psychologist	3 (14.3)	10 (17.5)	1.000
Case manager	1 (4.8)	4 (7.0)	1.000
Chaplain	2 (9.5)	3 (5.3)	0.607
Patient advocate	2 (9.5)	5 (8.8)	1.000
Other	1 (4.8)	3 (5.3)	1.000
Do not know	3 (14.3)	7 (12.3)	1.000
7. Do you believe that once you make an important medical decision, for example, to be put on a respirator, a machine that breathes for you, that you would be able to change your mind?			0.609
Yes, definitely	6 (28.6)	13 (22.8)	
Very probably	3 (14.3)	8 (14.0)	
Probably	5 (23.8)	20 (35.1)	
Probably not	6 (28.6)	9 (15.8)	
Do not know	1 (4.8)	7 (12.3)	
8. Do you think your doctor or the hospital will respect your wishes, that is, do what you want about medical care?			0.221
Yes, definitely	13 (61.9)	27 (47.4)	

© 2020 Friebert S et al. *JAMA Network Open.*

**eTable 5. (continued) Frequencies of Adolescent Responses for Survey by Poverty at Session 1 (N=78[a])**			
**Question**	**Poverty (N=21)**	**Non-Poverty (N=57)**	**P-value[b]**
	**N (%)**	**N (%)**	
Very probably	7 (33.3)	12 (21.1)	
Probably	1 (4.8)	11 (19.3)	
Probably not	0 (0.0)	4 (7.0)	
Do not know	0 (0.0)	3 (5.3)	
9. Do you think your parent/guardian/surrogate understands your wishes?			0.931
Yes, definitely	10 (50.0)	30 (52.6)	
Very probably	4 (20.0)	9 (15.8)	
Probably	5 (25.0)	10 (17.5)	
Probably not	1 (5.0)	4 (7.0)	
Definitely no	0 (0.0)	1 (1.8)	
Do not know	0 (0.0)	3 (5.3)	
10. Do you think your parent/guardian/surrogate will respect your wishes, that is, do what you want about your medical care?			0.666
Yes, definitely	9 (42.9)	31 (54.4)	
Very probably	6 (28.6)	11 (19.3)	
Probably	5 (23.8)	9 (15.8)	
Probably not	1 (4.8)	3 (5.3)	
Do not know	0 (0.0)	3 (5.3)	
11. How often has death and dying been talked about in your family?			0.813
Very often	0 (0.0)	2 (3.5)	
Often	3 (14.3)	9 (15.8)	
Occasionally	8 (38.1)	18 (31.6)	
Rarely	9 (42.9)	21 (36.8)	
Never	0 (0.0)	5 (8.8)	
Do not know	1 (4.8)	2 (3.5)	
12. How comfortable are you talking about death?			0.482
Very comfortable	4 (19.0)	13 (22.8)	
Somewhat comfortable	9 (42.9)	23 (40.4)	
Neither comfortable or uncomfortable	3 (14.3)	13 (22.8)	
Not very comfortable	1 (4.8)	5 (8.8)	
Not at all comfortable	3 (14.3)	2 (3.5)	
Do not know	1 (4.8)	1 (1.8)	
13. How likely are you to?			
13a. Attend funerals or memorial services when a loved one, friend or classmate dies			0.392
Very likely	19 (90.5)	40 (70.2)	

© 2020 Friebert S et al. *JAMA Network Open.*

eTable 5. (continued) Frequencies of Adolescent Responses for Survey by Poverty at Session 1 (N=78[a])			
**Question**	**Poverty (N=21)**	**Non-Poverty (N=57)**	**P-value[b]**
	**N (%)**	**N (%)**	
Somewhat likely	1 (4.8)	11 (19.3)	
Neither likely or unlikely	1 (4.8)	1 (1.8)	
Not very likely	0 (0.0)	1 (1.8)	
Not at all likely	0 (0.0)	2 (3.5)	
Do not know	0 (0.0)	2 (3.5)	
13b. Avoid medical checkups because you are afraid the doctor will find "something serious"			0.035
Very likely	0 (0.0)	2 (3.5)	
Somewhat likely	4 (19.0)	7 (12.3)	
Neither likely or unlikely	2 (9.5)	1 (1.8)	
Not very likely	0 (0.0)	11 (19.3)	
Not at all likely	14 (66.7)	36 (63.2)	
Do not know	1 (4.8)	0 (0.0)	
13c. Speak freely to loved ones about death and dying			0.173
Very likely	3 (14.3)	14 (24.6)	
Somewhat likely	8 (38.1)	20 (35.1)	
Neither likely or unlikely	7 (33.3)	10 (17.5)	
Not very likely	0 (0.0)	7 (12.3)	
Not at all likely	2 (9.5)	6 (10.5)	
Do not know	1 (4.8)	0 (0.0)	
13d. Visit or telephone a friend or relative who has recently lost a loved one in order to see how they are doing			1.000
Very likely	16 (76.2)	41 (71.9)	
Somewhat likely	4 (19.0)	12 (21.1)	
Neither likely or unlikely	1 (4.8)	2 (3.5)	
Not very likely	0 (0.0)	2 (3.5)	
13e. Preplan your own funeral, for example, choose someone to speak or choose the music you would want			0.429
Very likely	8 (38.1)	18 (31.6)	
Somewhat likely	4 (19.0)	11 (19.3)	
Neither likely or unlikely	6 (28.6)	7 (12.3)	
Not very likely	1 (4.8)	8 (14.0)	
Not at all likely	1 (4.8)	9 (15.8)	
Do not know	1 (4.8)	4 (7.0)	
14. How afraid, if at all, are you of?			
14a. Dying from a long term illness			0.405
Very afraid	3 (14.3)	6 (10.5)	
Somewhat afraid	6 (28.6)	19 (33.3)	

© 2020 Friebert S et al. *JAMA Network Open.*

**eTable 5. (continued) Frequencies of Adolescent Responses for Survey by Poverty at Session 1 (N=78[a])**			
**Question**	**Poverty (N=21)**	**Non-Poverty (N=57)**	**P-value[b]**
	**N (%)**	**N (%)**	
Neither afraid nor not afraid	5 (23.8)	4 (7.0)	
Not very afraid	3 (14.3)	13 (22.8)	
Not at all afraid	4 (19.0)	12 (21.1)	
Do not know	0 (0.0)	3 (5.3)	
14b. Dying suddenly, such as an accident, or being killed:			0.430
Very afraid	5 (23.8)	9 (15.8)	
Somewhat afraid	7 (33.3)	18 (31.6)	
Neither afraid nor not afraid	2 (9.5)	11 (19.3)	
Not very afraid	2 (9.5)	11 (19.3)	
Not at all afraid	4 (19.0)	8 (14.0)	
Do not know	1 (4.8)	0 (0.0)	
14c. Dying alone			0.142
Very afraid	9 (42.9)	15 (26.3)	
Somewhat afraid	4 (19.0)	10 (17.5)	
Neither afraid nor not afraid	3 (14.3)	9 (15.8)	
Not very afraid	0 (0.0)	10 (17.5)	
Not at all afraid	4 (19.0)	13 (22.8)	
Do not know	1 (4.8)	0 (0.0)	
14d. Dying in an institution such as a nursing home or hospital			0.135
Very afraid	3 (14.3)	10 (17.5)	
Somewhat afraid	3 (14.3)	9 (15.8)	
Neither afraid nor not afraid	6 (28.6)	8 (14.0)	
Not very afraid	1 (4.8)	15 (26.3)	
Not at all afraid	6 (28.6)	14 (24.6)	
Do not know	2 (9.5)	1 (1.8)	
14e. Dying painfully			0.150
Very afraid	10 (47.6)	17 (29.8)	
Somewhat afraid	6 (28.6)	19 (33.3)	
Neither afraid nor not afraid	1 (4.8)	5 (8.8)	
Not very afraid	0 (0.0)	9 (15.8)	
Not at all afraid	3 (14.3)	7 (12.3)	
Do not know	1 (4.8)	0 (0.0)	
15. How strongly do you agree or disagree that?			
15a. Dying is an important part of life			0.003
Strongly agree	3 (14.3)	29 (50.9)	
Agree	10 (47.6)	20 (35.1)	
Neither agree or disagree	6 (28.6)	3 (5.3)	

© 2020 Friebert S et al. *JAMA Network Open.*

eTable 5. (continued) Frequencies of Adolescent Responses for Survey by Poverty at Session 1 (N=78[a])			
Question	Poverty (N=21)	Non-Poverty (N=57)	P-value[b]
	N (%)	N (%)	
Disagree	1 (4.8)	4 (7.0)	
Strongly disagree	1 (4.8)	0 (0.0)	
Do not know	0 (0.0)	1 (1.8)	
15b. If someone could tell me when I would die, I would want to know.			0.851
Strongly agree	6 (28.6)	9 (15.8)	
Agree	4 (19.0)	10 (17.5)	
Neither agree or disagree	3 (14.3)	7 (12.3)	
Disagree	2 (9.5)	8 (14.0)	
Strongly disagree	5 (23.8)	19 (33.3)	
Do not know	1 (4.8)	4 (7.0)	
16. When you think about death and dying, how concerned are you that?			
16a. The family's money won't last:			0.137
Very concerned	1 (4.8)	2 (3.5)	
Concerned	4 (19.0)	16 (28.1)	
Neither concerned nor unconcerned	5 (23.8)	14 (24.6)	
Not concerned	4 (19.0)	9 (15.8)	
Not at all concerned	3 (14.3)	15 (26.3)	
Do not know	4 (19.0)	1 (1.8)	
16b. I will be a burden to, or overload, my family or friends.			0.574
Very concerned	2 (9.5)	11 (19.3)	
Concerned	10 (47.6)	15 (26.3)	
Neither concerned nor unconcerned	1 (4.8)	6 (10.5)	
Not concerned	5 (23.8)	14 (24.6)	
Not at all concerned	2 (9.5)	9 (15.8)	
Do not know	1 (4.8)	2 (3.5)	
17. Which of the following health problems, if any, do you think are worse than death? Select all that apply.[f]			
Living with great pain	7 (33.3)	25 (43.9)	0.402
Total physical dependency on others, for example, being in a wheelchair	2 (9.5)	20 (35.1)	0.045
Not being able to communicate my wishes and/or care to family members, for example, being in a coma Checked	15 (71.4)	31 (54.4)	0.175
None are worse than death	4 (19.0)	17 (29.8)	0.402
18. How important would each of the following be to you if you were dealing with your own dying?			
18a. Family/friends visiting you			0.876
Very important	18 (85.7)	49 (86.0)	

© 2020 Friebert S et al. *JAMA Network Open.*

**eTable 5. (continued) Frequencies of Adolescent Responses for Survey by Poverty at Session 1 (N=78[a])**			
**Question**	**Poverty (N=21)**	**Non-Poverty (N=57)**	**P-value[b]**
	**N (%)**	**N (%)**	
Somewhat important	2 (9.5)	6 (10.5)	
Neither important nor unimportant	1 (4.8)	1 (1.8)	
Do not know	0 (0.0)	1 (1.8)	
18b. Being able to stay in your own home			0.385
Very important	5 (23.8)	22 (38.6)	
Somewhat important	10 (47.6)	22 (38.6)	
Neither important nor unimportant	3 (14.3)	4 (7.0)	
Not very important	2 (9.5)	7 (12.3)	
Not at all important	0 (0.0)	2 (3.5)	
Do not know	1 (4.8)	0 (0.0)	
18c. Honest answers from your doctor			0.469
Very important	20 (95.2)	56 (98.2)	
Somewhat important	1 (4.8)	1 (1.8)	
18d. Comfort from church services or persons such as a minister, priest, imam, or rabbi			0.012
Very important	4 (19.0)	26 (45.6)	
Somewhat important	10 (47.6)	12 (21.1)	
Neither important nor unimportant	4 (19.0)	4 (7.0)	
Not very important	0 (0.0)	5 (8.8)	
Not at all important	2 (9.5)	10 (17.5)	
Do not know	1 (4.8)	0 (0.0)	
18e. Planning your own funeral			0.491
Very important	4 (19.0)	12 (21.1)	
Somewhat important	6 (28.6)	21 (36.8)	
Neither important nor unimportant	8 (38.1)	10 (17.5)	
Not very important	2 (9.5)	9 (15.8)	
Not at all important	0 (0.0)	3 (5.3)	
Do not know	1 (4.8)	2 (3.5)	
18f. Being able to complete an advance directive that would let loved ones know your wishes, if you were unable to speak for yourself.			0.121
Very important	16 (76.2)	27 (47.4)	
Somewhat important	4 (19.0)	23 (40.4)	
Neither important nor unimportant	0 (0.0)	4 (7.0)	
Do not know	1 (4.8)	3 (5.3)	
18g. Fulfilling personal goals/pleasures			0.123
Very important	11 (52.4)	43 (75.4)	
Somewhat important	9 (42.9)	12 (21.1)	

© 2020 Friebert S et al. *JAMA Network Open.*

**eTable 5. (continued) Frequencies of Adolescent Responses for Survey by Poverty at Session 1 (N=78[a])**			
**Question**	**Poverty (N=21)**	**Non-Poverty (N=57)**	**P-value[b]**
	**N (%)**	**N (%)**	
Neither important nor unimportant	1 (4.8)	1 (1.8)	
Not very important	0 (0.0)	1 (1.8)	
18h. Reviewing your life history with your family			0.845
Very important	8 (38.1)	25 (43.9)	
Somewhat important	6 (28.6)	17 (29.8)	
Neither important nor unimportant	5 (23.8)	8 (14.0)	
Not very important	2 (9.5)	6 (10.5)	
Do not know	0 (0.0)	1 (1.8)	
18i. Having health care professionals visit you at your home			0.586
Very important	6 (28.6)	18 (31.6)	
Somewhat important	8 (38.1)	25 (43.9)	
Neither important nor unimportant	5 (23.8)	12 (21.1)	
Not very important	1 (4.8)	1 (1.8)	
Not at all important	0 (0.0)	1 (1.8)	
Do not know	1 (4.8)	0 (0.0)	
18j. Understanding your treatment choices			0.363
Very important	21 (100.0)	50 (87.7)	
Somewhat important	0 (0.0)	5 (8.8)	
Do not know	0 (0.0)	2 (3.5)	
19. How important are each of the following is to you when you think about dying?			
19a. Being physically comfortable			0.264
Very important	15 (71.4)	33 (57.9)	
Somewhat important	4 (19.0)	20 (35.1)	
Neither important nor unimportant	1 (4.8)	2 (3.5)	
Not very important	1 (4.8)	0 (0.0)	
Not at all important	0 (0.0)	2 (3.5)	
19b. Being free from pain			0.410
Very important	15 (71.4)	35 (61.4)	
Somewhat important	4 (19.0)	13 (22.8)	
Neither important nor unimportant	0 (0.0)	5 (8.8)	
Not very important	2 (9.5)	1 (1.8)	
Not at all important	0 (0.0)	2 (3.5)	
Do not know	0 (0.0)	1 (1.8)	
19c. Saying everything I want to say to people in my family			0.244
Very important	14 (70.0)	47 (82.5)	
Somewhat important	5 (25.0)	9 (15.8)	
Neither important nor unimportant	1 (5.0)	0 (0.0)	

© 2020 Friebert S et al. *JAMA Network Open.*

**eTable 5. (continued) Frequencies of Adolescent Responses for Survey by Poverty at Session 1 (N=78[a])**			
**Question**	**Poverty (N=21)**	**Non-Poverty (N=57)**	**P-value[b]**
	**N (%)**	**N (%)**	
Not at all important	0 (0.0)	1 (1.8)	
19d. Being at peace spiritually			0.095
Very important	13 (61.9)	45 (78.9)	
Somewhat important	5 (23.8)	9 (15.8)	
Neither important nor unimportant	0 (0.0)	2 (3.5)	
Not very important	1 (4.8)	1 (1.8)	
Do not know	2 (9.5)	0 (0.0)	
19e. Not being a burden to loved ones			0.464
Very important	13 (61.9)	39 (68.4)	
Somewhat important	5 (23.8)	10 (17.5)	
Neither important nor unimportant	1 (4.8)	4 (7.0)	
Not very important	0 (0.0)	3 (5.3)	
Do not know	2 (9.5)	1 (1.8)	
19f. Knowing how to say good bye			0.880
Very important	16 (76.2)	42 (73.7)	
Somewhat important	3 (14.3)	8 (14.0)	
Neither important nor unimportant	1 (4.8)	3 (5.3)	
Not very important	1 (4.8)	1 (1.8)	
Do not know	0 (0.0)	3 (5.3)	
19g. Having a sense of my own worth or value			0.445
Very important	12 (57.1)	36 (63.2)	
Somewhat important	6 (28.6)	17 (29.8)	
Neither important nor unimportant	1 (4.8)	3 (5.3)	
Do not know	2 (9.5)	1 (1.8)	
19h. Being off machines that extend life, such as life support.			0.458
Very important	6 (28.6)	17 (29.8)	
Somewhat important	10 (47.6)	14 (24.6)	
Neither important nor unimportant	3 (14.3)	17 (29.8)	
Not very important	1 (4.8)	3 (5.3)	
Not at all important	0 (0.0)	2 (3.5)	
Do not know	1 (4.8)	4 (7.0)	
19i. Dying a natural death			0.173
Very important	6 (28.6)	19 (33.3)	
Somewhat important	11 (52.4)	12 (21.1)	
Neither important nor unimportant	3 (14.3)	15 (26.3)	
Not very important	1 (4.8)	5 (8.8)	
Not at all important	0 (0.0)	2 (3.5)	

© 2020 Friebert S et al. *JAMA Network Open.*

eTable 5. (continued) Frequencies of Adolescent Responses for Survey by Poverty at Session 1 (N=78[a])			
Question	Poverty (N=21)	Non-Poverty (N=57)	P-value[b]
	N (%)	N (%)	
Do not know	0 (0.0)	4 (7.0)	
20. If death were likely to happen in the next few weeks, and you could choose where to die, where would you MOST want to die?			0.546
At home without hospice	9 (42.9)	14 (24.6)	
At home in hospice	4 (19.0)	16 (28.1)	
In a hospital	1 (4.8)	8 (14.0)	
No preference	3 (14.3)	8 (14.0)	
Do not know	4 (19.0)	11 (19.3)	
21. Below are some statements related to pain near the end-of-life that have been expressed by people. How strongly do you agree or disagree with each statement?			
21a. I am afraid the doctor may not believe I am in pain and treat my pain			0.454
Strongly agree	0 (0.0)	2 (3.5)	
Agree	6 (28.6)	7 (12.3)	
Neither agree or disagree	4 (19.0)	6 (10.5)	
Disagree	6 (28.6)	23 (40.4)	
Strongly disagree	4 (19.0)	14 (24.6)	
Do not know	1 (4.8)	5 (8.8)	
21b. I would only take pain medicines when the pain is severe			0.120
Strongly agree	9 (42.9)	16 (28.1)	
Agree	6 (28.6)	21 (36.8)	
Neither agree or disagree	0 (0.0)	9 (15.8)	
Disagree	5 (23.8)	10 (17.5)	
Strongly disagree	1 (4.8)	0 (0.0)	
Do not know	0 (0.0)	1 (1.8)	
21c. I am afraid I will become addicted to the pain medicines over time			0.805
Strongly agree	0 (0.0)	3 (5.3)	
Agree	6 (28.6)	9 (15.8)	
Neither agree or disagree	1 (4.8)	4 (7.0)	
Disagree	7 (33.3)	17 (29.8)	
Strongly disagree	6 (28.6)	21 (36.8)	
Do not know	1 (4.8)	3 (5.3)	
21d. I would take the lowest amount of medicine possible to save larger doses for later when the pain is worse			0.877
Strongly agree	4 (19.0)	11 (19.3)	
Agree	8 (38.1)	22 (38.6)	
Neither agree or disagree	4 (19.0)	8 (14.0)	

© 2020 Friebert S et al. *JAMA Network Open.*

**eTable 5. (continued) Frequencies of Adolescent Responses for Survey by Poverty at Session 1 (N=78[a])**			
**Question**	**Poverty (N=21)**	**Non-Poverty (N=57)**	**P-value[b]**
	**N (%)**	**N (%)**	
Disagree	4 (19.0)	7 (12.3)	
Strongly disagree	1 (4.8)	7 (12.3)	
Do not know	0 (0.0)	2 (3.5)	
21e. I am afraid I would be given too much pain medicine			0.214
Strongly agree	0 (0.0)	6 (10.5)	
Agree	6 (28.6)	8 (14.0)	
Neither agree or disagree	4 (19.0)	4 (7.0)	
Disagree	7 (33.3)	22 (38.6)	
Strongly disagree	4 (19.0)	14 (24.6)	
Do not know	0 (0.0)	3 (5.3)	
22. Have you heard of hospice services?			0.050
Yes	11 (52.4)	43 (75.4)	
No	10 (47.6)	14 (24.6)	
23. How did you learn about hospice services? (N=56)			1.000
I know someone who used hospice services	6 (54.5)	21 (48.8)	
I heard from a health care professional	1 (9.1)	3 (7.0)	
I read literature/newspaper/TV/radio/other media	1 (9.1)	6 (14.0)	
I heard from others	3 (27.3)	13 (30.2)	
24. If you were dying, would you want hospice support? (N=56)			0.446
Yes	3 (27.3)	21 (48.8)	
No	1 (9.1)	5 (11.6)	
Don't know/not sure	7 (63.6)	17 (39.5)	
25. Do you consider yourself religious/spiritual?			0.073
Very religious/spiritual	3 (14.3)	18 (31.6)	
Somewhat religious/spiritual	9 (42.9)	28 (49.1)	
Not very religious/spiritual	5 (23.8)	4 (7.0)	
Not religious/spiritual	3 (14.3)	7 (12.3)	
Declined	1 (4.8)	0 (0.0)	
26. How often do you attend religious or spiritual services? (N=69)			0.650
Always	1 (5.9)	5 (10.0)	
Very often	2 (11.8)	14 (28.0)	
Sometimes	10 (58.8)	21 (42.0)	
Rarely	3 (17.6)	8 (16.0)	
Never	1 (5.9)	2 (4.0)	
27. How often does religion or spirituality help you face your fears or do what you were afraid to do? (N=69)			0.330
Everyday	2 (11.8)	16 (32.0)	

© 2020 Friebert S et al. *JAMA Network Open.*

**eTable 5. (continued) Frequencies of Adolescent Responses for Survey by Poverty at Session 1 (N=78[a])**			
**Question**	**Poverty (N=21)**	**Non-Poverty (N=57)**	**P-value[b]**
	**N (%)**	**N (%)**	
A few times a week	7 (41.2)	10 (20.0)	
A few times a month	4 (23.5)	13 (26.0)	
Rarely	3 (17.6)	8 (16.0)	
Never	1 (5.9)	3 (6.0)	
30. HOW MANY NIGHTS did you stay in a hospital?			0.413
0	19 (90.5)	50 (87.7)	
1-2	0 (0.0)	1 (1.8)	
3-5	0 (0.0)	4 (7.0)	
6-10	2 (9.5)	1 (1.8)	
>20	0 (0.0)	1 (1.8)	
31. How healthy are you feeling right now?			0.671
Excellent health	4 (19.0)	18 (31.6)	
Very good health	10 (47.6)	20 (35.1)	
Good health	3 (14.3)	11 (19.3)	
Fair health	3 (14.3)	6 (10.5)	
Poor health	1 (4.8)	1 (1.8)	
Declined	0 (0.0)	1 (1.8)	
32a. Is your mother alive?			1.000
Yes	21 (100.0)	56 (98.2)	
No	0 (0.0)	1 (1.8)	
32b. Is your father alive?			0.794
Yes	19 (90.5)	52 (91.2)	
No	1 (4.8)	4 (7.0)	
Do not know	1 (4.8)	1 (1.8)	

[a]: Two unknown poverty cases was excluded.
[b]: Pearson chi-square test or Fisher' exact test.
[c]: Bonferroni corrected significant level is 0.05/8=0.006.
[d]: Bonferroni corrected significant level is 0.05/4=0.013.
[e]: Bonferroni corrected significant level is 0.05/10=0.005.
[f]: Bonferroni corrected significant level is 0.05/4=0.013.

© 2020 Friebert S et al. *JAMA Network Open.*

eTable 6. The Congruence on End of Life Needs for Adolescents Living with Cancer Between Adolescents' and Families' Perception of What They Thought their Adolescents Preferred (N=80 dyads)		
**Question[a]**	**Congruence N (%)**	**PABAK[b]**
How comfortable are you talking about death?		
Comfortable	23 (28.8)	0.05
Otherwise	19 (23.8)	
When you think about death and dying, how concerned are you that you will be a burden to, or overload, my family or friends?		
Concerned	20 (25.0)	0.08
Otherwise	23 (28.8)	
How strongly do you agree or disagree that if someone could tell me when I would die, I would want to know?		
Agree	14 (17.5)	0.18
Otherwise	33 (41.3)	
I am afraid the doctor may not believe I am in pain and treat my pain.		
Agree	6 (7.5)	0.48
Otherwise	53 (66.3)	
I would only take pain medicines when the pain is severe.		
Agree	44 (55.0)	0.33
Otherwise	9 (11.3)	
I am afraid I will become addicted to the pain medicines over time.		
Agree	10 (12.5)	0.25
Otherwise	40 (50.0)	
I would take the lowest amount of medicine possible to save larger doses for later when the pain is worse.		
Agree	33 (41.3)	0.18
Otherwise	14 (17.5)	

[a]:"Comfortable" included Very comfortable/Somewhat comfortable; "Otherwise" included Neither comfortable nor uncomfortable/Not very comfortable/Not at all comfortable/Do not know.
"Concerned" included Very concerned/Concerned; "Otherwise" included Neither concerned nor unconcerned/Not concerned/Not at all concerned/Do not know.
"Agree" included Strongly agree/Agree; "Otherwise" included Neither agree nor disagree/Disagree/ Strongly disagree/Do not know.
[b]:PABAK: The prevalence adjusted bias adjusted Kappa: Less than 0.40 = poor; Between 0.40 – 0.59 = fair, Between 0.60 – 0.74 = good; Between 0.75 – 1.00 = excellent.

© 2020 Friebert S et al. *JAMA Network Open.*
